# Inhibition of RUNX2 Transcriptional Activity Blocks the Proliferation, Migration and Invasion of Epithelial Ovarian Carcinoma Cells

**DOI:** 10.1371/journal.pone.0074384

**Published:** 2013-10-04

**Authors:** Zhi-Qiang Wang, Mamadou Keita, Magdalena Bachvarova, Stephane Gobeil, Chantale Morin, Marie Plante, Jean Gregoire, Marie-Claude Renaud, Alexandra Sebastianelli, Xuan Bich Trinh, Dimcho Bachvarov

**Affiliations:** 1 Department of Molecular Medicine, Laval University, Québec (Québec), Canada; 2 Centre de recherche du CHU de Québec, L'Hôtel-Dieu de Québec, Québec (Québec), Canada; 3 Centre de recherche du CHU de Québec, CHUL, Québec (Québec), Canada; 4 Department of Obstetrics and Gynecology, Laval University, Québec (Québec), Canada; 5 Department of Gynecological Oncology, Antwerp University Hospital, Antwerp, Belgium; University of Quebec at Trois-Rivieres, Canada

## Abstract

Previously, we have identified the RUNX2 gene as hypomethylated and overexpressed in post-chemotherapy (CT) primary cultures derived from serous epithelial ovarian cancer (EOC) patients, when compared to primary cultures derived from matched primary (prior to CT) tumors. However, we found no differences in the RUNX2 methylation in primary EOC tumors and EOC omental metastases, suggesting that DNA methylation-based epigenetic mechanisms have no impact on RUNX2 expression in advanced (metastatic) stage of the disease. Moreover, RUNX2 displayed significantly higher expression not only in metastatic tissue, but also in high-grade primary tumors and even in low malignant potential tumors. Knockdown of the RUNX2 expression in EOC cells led to a sharp decrease of cell proliferation and significantly inhibited EOC cell migration and invasion. Gene expression profiling and consecutive network and pathway analyses confirmed these findings, as various genes and pathways known previously to be implicated in ovarian tumorigenesis, including EOC tumor invasion and metastasis, were found to be downregulated upon RUNX2 suppression, while a number of pro-apoptotic genes and some EOC tumor suppressor genes were induced.

Taken together, our data are indicative for a strong oncogenic potential of the RUNX2 gene in serous EOC progression and suggest that RUNX2 might be a novel EOC therapeutic target. Further studies are needed to more completely elucidate the functional implications of RUNX2 and other members of the RUNX gene family in ovarian tumorigenesis.

## Introduction

Epithelial ovarian cancer (EOC) is a disease that is responsible for more cancer deaths among women in the Western world than all other gynecologic malignancies [1]. EOC lethality primarily stems from the inability to detect the disease at an early, organ-confined stage, and the lack of effective therapies for advanced-stage disease [2]. Indeed, despite treatment improvements [3], the majority of women continue to present at advanced stages with a 5-year survival rate of less than 40%. The currently established therapy of ovarian cancer includes radical surgical tumor debulking and subsequent platinum plus paclitaxel–based chemotherapy (CT). However, a significant risk of recurrence and resistance to therapy remains and when this occurs, ovarian cancer is currently incurable [4]. So there is a need for new therapeutic targets and a better understanding of the mechanisms involved in the spread of ovarian carcinoma.

It is well established that cancer invasion and metastasis still represent the major causes of the failure of cancer treatment. Approximately 70% of patients with advanced-stage EOC have widespread intraperitoneal metastases, including the formation of malignant serous effusions within the peritoneal cavity [1]. Pleural effusions constitute the most frequent site of distant metastasis (FIGO stage IV disease). Unlike the majority of solid tumors, particularly at the primary site, cancer cells in effusions are not amenable to surgical removal, and failure in their eradication is one of the main causes of treatment failure. Thus, management of the metastatic disease becomes a crucial problem for the treatment of EOC. One possible way to resolve this problem is to target metastasis-specific pathways with novel therapies. Hence, focused identification of novel pro-metastatic target pathways and molecules could enhance the chances of discovering new and effective therapies.

Recently, the importance of epigenetic perturbation of gene regulation in cancer [5], including EOC [6], has begun to be more fully appreciated. The most studied epigenetic alteration is DNA methylation, the addition of a methyl moiety to the cytosine-5 position within the context of a CpG dinucleotide, mediated by DNA methyltransferases [5]. In cancer, promoter hypermethylation often leads to inactivation of different tumor-suppressing genes and is associated with many important pathways involved in cancer progression [7] and the development of resistance to chemotherapy (CT) [8]. The role of DNA hypomethylation in carcinogenesis is less studied. Similar to other malignancies, aberrant DNA methylation, including global hypomethylation of heterochromatin and local CpG island methylation, occurs in EOC and contributes to ovarian tumorigenesis and mechanisms of chemoresistance [6].

Using an epigenomic approach (methylated DNA immunoprecipitation coupled to CpG island tiling arrays) we have recently shown that DNA hypermethylation occurs in less invasive/early stages of ovarian tumorigenesis, while advanced disease was associated with DNA hypomethylation of a number of oncogenes, implicated in cancer progression, invasion/metastasis and probably chemoresistance [9]. In this study we have also shown, that the RUNX1 and RUNX2 transcription factors were hypomethylated and overexpressed in primary cell cultures (PCCs) derived from post-CT tumors of two serous EOC patients, when compared to PCCs derived from matched primary (pre-CT) tumors [9].

The RUNX gene family comprises the RUNX1, RUNX2 and RUNX3 transcription factors, each of which is capable of forming heterodimers with the common CBFβ cofactor (a non-DNA-binding partner), as components of the core-binding factor (CBF) complex [10]. These transcription factors can activate or repress transcription of key regulators of growth, survival and differentiation pathways [11]. Although the RUNX family members share considerable amino acid identity and display some overlapping functions, they nevertheless appear to have distinct biological functions during development, with each of the three corresponding RUNX knockout mice displaying highly distinct phenotypic abnormalities. RUNX1 is essential for definitive hematopoiesis, megakaryocyte maturation, T- and B-cell lineages and neuronal development [12], [13]. RUNX2 is essential for osteogenesis [14]. RUNX3 has essential roles in neurogenesis [15], TGF-β signaling and dendritic cell maturation [16]. RUNX factors are increasingly linked to various human cancers, as they could function both as tumor suppressor genes (TSGs) and dominant oncogenes in a context-dependent manner (reviewed in [17]). RUNX3 is generally considered as a TSG in human neoplasia as a multitude of epithelial cancers exhibit inactivation of RUNX3 [18], including ovarian carcinoma [19], although oncogenic function of RUNX3 in EOC was also suggested [20]. The importance of RUNX1 in hematopoiesis and its TSG function in leukemia are well established [21], although RUNX1 gene amplifications and gain-of-RUNX1 function mutations have been postulated to have leukemogenic effects [22], [23]. Similarly, recent studies in solid tumors present contrasting roles of RUNX1 as either TSG or oncogene (reviewed in [21]). The implication of RUNX1 in EOC tumorigenesis is currently unknown, although it was shown that in conjunction with some matrix metalloproteinases (MMP-2 and -9) RUNX1 could contribute to the invasive stage of endometrial and ovarian endometrioid carcinomas [24]. We have recently shown that RUNX1 is significantly overexpressed in serous EOC tumors, although DNA hypomethylation was not significantly associated with its induction in advanced (metastatic) stage of the disease [25]. Moreover, RUNX1 expression was associated with increased EOC cell proliferation, migration and invasion, suggesting for a strong oncogenic potential of the RUNX1 gene in EOC progression [25].

Similar to RUNX1, the strongest evidence for a pro-oncogenic function for RUNX2 comes from studies in lymphoma/leukemia models [26]; however RUNX2 was also shown to play a role in invasive bone [27], breast [28], prostate [29], thyroid [30] and pancreatic cancer [31]. Lately, RUNX2 expression was also associated with EOC tumor progression and poor prognosis [32]. This prompted us to investigate if RUNX2 is induced due to hypomethylation in advanced EOC and whether the RUNX2 gene is functionally implicated in EOC tumorigenesis, including disease progression and response to treatment. Here we show that, similar to RUNX1, the RUNX2 gene is functionally involved in EOC cell proliferation, migration and invasion. However, we also demonstrate that RUNX1 and RUNX2 employ molecular mechanisms in EOC dissemination that are specific for each gene.

## Materials and Methods

### Ethics statement

This study obtained approval from the Clinical Research Ethics Committee of the Hotel-Dieu de Quebec Hospital and patients gave written consent for tissue collection and analyses.

### Patients and tissue specimens

Snap frozen and formalin-fixed paraffin-embedded (FFPE) tissues of 117 serous EOC tumors were provided by the Banque de tissus et de données of the Réseau de recherche sur le cancer of the Fonds de recherche du Québec - Santé at the Hotel-Dieu de Quebec Hospital, Quebec, Canada, which is affiliated with the Canadian Tumor Repository Network. These clinical specimens included 13 borderline, or low-malignant potential (LMP) tumors, 52 high-grade adenocarcinomas and 52 omental metastases. None of the patients received chemotherapy before surgery (see Table 1 and Table S1 for detailed clinicopathological characteristics). All tumors were histologically classified according to the criteria defined by the World Health Organization [33]. The CT treatment was completed for all patients and the response to treatment was known. Disease progression was evaluated following the guidelines of the Gynecology Cancer Intergroup [33]. Progression free survival (PFS) was defined as the time from surgery to the first observation of disease progression, recurrence or death. Thirteen normal ovarian samples and 13 normal uterine smooth muscle samples were derived from women subjected to hysterectomy with oophorectomy due to non-ovarian pathologies.

**Table 1:** Patients' characteristics.

Variable	Range	n/total	%
Age (years)	<50	18/130	14.0
	50–69	66/130	50.9
	>70	46/130	35.1
Median age	64		
Tissue/tumor type	Normal	13/130	10.0
	LMP	13/130	10.0
	High-grade	52/130	40.0
	OM	52/130	40.0
Stage	III (A, B and C)	69/130	53.0
	IV	30/130	23.0
PFS (months)*	0–6	43/99	43.4
	7–24	35/99	35.4
	>25	21/99	21.2

* Extended follow-up, including PFS values, were available for 99 patients.

### Cell cultures

The EOC cell lines OVCAR3, SKOV3 and C13 were purchased from American Tissue Type Collection (Manassas, VA); OV-90, OV2008, TOV-112 and TOV-21 cell lines were a kind gift from Dr. Anne-Marie Mes-Masson (Montreal University) [34], while A2780s and A2780cp cell lines were a kind gift from Dr. Benjamin Tsang (Ottawa University) [35]. The cell lines were passaged in different culture media supplemented with 10% fetal bovine serum, as described previously [36].

### Bisulfite sequencing PCR (BSP) analysis

BSP analysis was performed, as previously described [37], [38]. Briefly, genomic DNAs from primary and metastatic EOC tumor specimens were isolated using the Qiagen DNeasy Blood and Tissue Kit. Bisulfite modification of genomic DNAs was done using the Methyl Detector kit (Active Motif, Carlsbad, CA). For BSP, a 285-bp fragment was amplified using primer pairs specific for bisulfite-modified sequences but not harboring CpGs, located at nt −2816 (GGTTTGGTTAAATGGGTTT) to nt −2531 (ACCCTTCCTCCATACACTACTC) upstream of the RUNX2 transcription start (ATG) codon. BSP primer selection was performed using the Methyl Primer Express Software v1.0 (Applied Biosystems). PCR was done for 35 cycles (94°C, 30 s; 60°C, 50 s; 72°C, 1 min). PCR products were sent for dideoxy-sequencing analysis at the Genomics Analysis Platform at Laval University (http://www.bioinfo.ulaval.ca/seq/en/).

### Tissue microarrays (TMAs) construction and immunohistochemistry (IHC)

TMAs were constructed, as previously described [39]. Briefly, one representative block of each ovarian tumor and normal ovarian tissue was selected for the preparation of the tissue arrays. Three 0.6 mm cores of tumor were taken from each tumor block and placed, 0.4 mm apart, on a recipient paraffin block using a commercial tissue arrayer (Beecher Instruments, Sun Prairie, WI). The cores were randomly placed on one of two recipient blocks to avoid IHC evaluation biases. Four micron thick sections were cut for the hematoxylin-eosin (HE) staining and IHC analyses.

IHC was performed, as previously described [37]–[39]. Briefly, 4 µm tissue sections were deparaffinized and then heated in an autoclave for 12 min to retrieve the antigenicity before blocking with endogenous peroxidase. Following treatment with 3% H_2_O_2_ for 10 min to quench the endogenous peroxidise activity, sections were incubated with anti-RUNX2 antibody (1∶100 dilution) (Santa Cruz Biotechnology; sc-101145) at room temperature for 2 hours. Sections were then incubated with a biotinylated secondary antibody (Dako, Carpinteria, CA) and then exposed to a streptavidin complex (Dako, Carpinteria, CA). Complete reaction was revealed by 3-3′ diaminobenzidine and slides were counterstained with hematoxylin. RUNX2 protein expression was assessed by semi-quantitative scoring of the intensity of staining and recorded as absent (0), weak (1+), moderate (2+) or strong (3+). The relationship between RUNX2 expression in serous ovarian carcinomas and normal ovarian tissues was evaluated by the Mann-Whitney test. A significant association was considered when p-value was below 0.05. A Kaplan Meier curve and the log-rank test were performed based on PFS values to test the effect of the intensity of RUNX2 (3, 2 versus 0, 1) on disease progression.

### Short hairpin RNA (shRNA) – mediated RUNX2 knockdown in EOC cells

The shRNA-mediated RUNX2 knockdown in SKOV3 and A2780s cells was done, as previously described [37], [38]. Briefly, a RUNX2 shRNA cloned into the pLKO.1-puro vector was retrieved from the Sigma Mission TRC human 1.5 shRNA library (clone number TRCN0000013655). Viral supernatants were generated by transfecting 293T cells with the shRNA construct and the packaging vectors psPAX2 and pMD2.G (Addgene, Cambridge, MA). The high-titer lentiviral supernatants in the presence of 8 µg/ml polybrene were used to infect SKOV3 and A2780s cells. Two days later, infected cells were treated with puromycin (0,5 µg/ml) for the selection of stably-transduced clones. The pLKO.1-puro vector encoding a scramble sequence not matching any mammalian sequence was used for the generation of mock-transduced (control) clones. Stable clones with inhibited RUNX2 expression were evaluated and validated by semi-quantitative RT-PCR and Western blot.

### Western blot analysis

Western blot analysis was performed as previously described [37], [38]. Briefly, protein lysates were prepared by resuspending cell pellets in Laemmli sample buffer containing 5% β-mercaptoethanol. Protein lysates were separated by 6 to 12% Tris-glycine gel electrophoresis and transferred onto a polyvinylidene difluoride membrane using a semi-dry apparatus (Bio-Rad Laboratories, Hercules, CA). The membrane was blocked with 5% nonfat dry milk in TBST (20 mmol/L Tris-HCl, 0.5 M NaCl, and 0.1% Tween 20), incubated with the anti-RUNX2 mouse monoclonal antibody (1∶500) (Santa Cruz Biotechnology) and anti-β-actin antibody (1∶5000) (Santa Cruz Biotechnology) at 4°C overnight. After 3×15 min washes with TBST (20 mmol/L Tris-HCl, 0.5 M NaCl, and 0.1% Tween 20) at room temperature, the membrane was incubated with horseradish peroxidase-conjugated secondary antibody and detected with ECL solution (Thermo Fisher Scientific, Waltham, MA).

### Cell proliferation assay using impedance measurement with the xCELLigence system

Cell proliferation (cell index) was checked by the xCELLigence Real-Time Cell Analyzer (RTCA) instrument, as previously described [38]. Cells were seeded in triplicate at 2×10^4^ cells/well in the E-Plate 16, a specialized 16-well plate used with the RTCA instrument. Each of the 16 wells on the E-Plate 16 contains an integral sensor electrode array so that cells inside each well can be monitored and assayed. Cell growth was monitored for 24 hours.

### Colony formation assay

Colony formation assay was performed, as previously described [37], [38]. Briefly, EOC cells were seeded at 500 cells per 60 mm culture dish. After 14 days, the dishes were washed twice in PBS, fixed with cold methanol, stained with Coomassie Blue (Sigma-Aldrich) for 5 min, washed with water and air dried. The number of colonies was determined by imaging with a Multimage™ Cabinet (Alpha Innotech Corporation, San Leandro, CA) and using AlphaEase Fc software.

### Cell migration and invasion assays

Cell migration and invasion assay were performed, as previously described [38]. Briefly, RUNX2 shRNA transduced, control (scramble shRNA) and intact SKOV3 and A2780s cells were seeded into the upper inserts of Boyden chambers (Costar, Cambridge, MA) in 0.1% FBS containing medium at a density of 2.5×10^4^ per well, and 600 µl of 1% FBS containing medium was placed in the lower chamber as a chemoattractant. After 24 h at 37°C in 5% CO_2_, the cells were fixed with cold methanol and stained with trypan blue solution. Cells on the upper surface of the filter were removed with cotton buds. Migrated cells on the underside of the filter were photographed and counted by phase contrast microscopy, by selecting 10 random fields per filter (at magnification×40). The experiments were performed in triplicate. Cell invasion was assayed in a similar way, as the 5-µm pore polycarbonate filters were coated with 40 µl of Matrigel™ at concentration of 0.5 mg/ml (BD Biosciences, Franklin Lakes, NJ). Here, 600 µl of NIH3T3 conditioned medium was added in the lower chamber as a chemoattractant. Differences between shRNA-RUNX2-transfected, vehicle-transfected and intact SKOV3 or A2780s cells were determined by a Student's t-test, where *p*<0.05 was considered significant.

### Flow cytometry

Flow cytometry analysis was performed, as previously described [38]. Briefly, 7.5×10^4^ SKOV3 cells were treated with 20 mM hydroxyurea (Sigma) for synchronization at the G1/S boundary. After 16 hours of incubation, cells were washed once with PBS, and resuspended in 1 ml of complete media (time 0). Then, cells were harvested by trypsinization at 0, 3, 6, 9, 24 and 48 h, washed three times with PBS, and fixed with ice–cold 95% ethanol overnight. Cells were washed with PBS (3×) and incubated with propidium iodide (50 µg/ml) (Sigma) in the dark at room temperature for 30 min. Flow cytometric analysis was performed on a Beckman Coulter EPICS XL-MCL analyzer. The cell cycle phase distribution was calculated from the resultant DNA using the cell QuesPro software.

### MTT (cytotoxicity) assay

The MTT cell proliferation assay (Sigma, St-Louis, MS, USA) was used to measure the cell growth inhibition effects of cisplatin and paclitaxel in SKOV3 cell clones suppressing RUNX2, as previously described [37]. Briefly, cell suspensions (at 2×10^4^ cells/ml) were transferred to 96-well plates in triplicates and incubated for 3 days with different drugs' concentrations (ranging between 1 nM and 100 µM). Then, 38 µl of 3-[4,5-dimethylthiazol-2-yl]-2,5-diphenyl-tetrazolium bromide (MTT, 5 mg/ml) was added to each well 4 h before the end of the incubation. After centrifugation and removing the supernatant, 200 µL of dimethyl sulphoxide (DMSO) were added to resolve the crystals and the optical density was measured by microplate reader at 595 nm.

### Gene expression profiling and data analysis

Gene expression analysis was carried out as previously described [36]. Briefly, total RNA was extracted from the shRNA-RUNX2 knockdown clone (cl-sh3) and the corresponding control (mock-transfected) SKOV3 clone. The quality of the RNA samples was examined by capillary electrophoresis using the Agilent 2100 Bioanalyzer (Agilent, Palo Alto, CA). Fluorescently labeled cRNA targets were generated from 0.5 µg of total RNA from each corresponding SKOV3 cell clone, using the Fluorescent Linear Amplification Kit (Agilent) and 10.0 mM Cyanine 3- or 5-labeled CTP (PerkinElmer, Boston, MA), and following user's manual. Cyanine labeled cRNA from the clone suppressing RUNX2 (cl-sh3) was mixed with the same amount of reverse-color cyanine-labeled cRNA from the corresponding control clone and hybridized on the Agilent Whole Human Genome microarrays, containing 44,000 genes. Array hybridization, washing, scanning, data extraction and analyses were performed as previously described [36]. Network analysis of the microarray data was completed using the Ingenuity Pathway Analysis (IPA) software (see http://www.Ingenuity.com). The microarray data have been deposited to GEO database with accession number GSE46477.

### Semi-quantitative duplex RT-PCR (sqRT-PCR)

Analysis of RUNX2 gene expression in stable RUNX2 knockdown clones (shRNA-RUNX2) and the corresponding mock-transfected SKOV3 and A2780 clones was performed by sqRT-PCR as previously described [36], [38]. The 18S ribosomal RNA gene was used as an internal standard. Comparative signal intensity was evaluated using the ImageJ software (http://rsb.info.nih.gov/ij/). Primers were designed for these loci with the sequences freely available from the Entrez Nucleotide database and the Primer3 algorithm for primer design (http://www-genome.wi.mit.edu/cgi-bin/primer/primer3_www.cgi).

### Quantitative PCR (qPCR)

For quantitative PCR validation of the gene erxpression data, total RNA was extracted by RNeasy Plus Mini Kit (QIAGEN) and cDNA was obtained by qScript™ cDNA SuperMix (Quanta BioSciences, Inc.). Primers were designed for these loci with the sequences freely available from the Entrez Nucleotide database and the Primer3 algorithm for primer design (http://www-genome.wi.mit.edu/cgi-bin/primer/primer3_www.cgi). The primers used for qPCR validation are listed in Table 2. PerfeCTa® SYBR® Green FastMix® (Quanta BioSciences, Inc.) was used used according to manufacturer's instructions. PCR reactions were performed on Rotor-Gene RG-3000 Real Time PCR System (GoIndustry DoveBid. com), with endogenous control 18S ribosomal RNA. PCR volume was 20 µL (36-well plate), and conditions were as follow: initial cycle 50°C, 2 min, 95°C, 15 min; 45 cycles at 95°C, 20 s , 60°C, 20 s and 72°C 20 s; final cycle 72°C 30 s . Data were analyzed by the Rotor-Gene software using the comparative ΔΔCt method. The relative copy number was calculated based on the target gene/18S RNA ratio.

**Table 2:** Primers for Quantitative PCR (qPCR).

Gene	Forward	Reverse
PCDH9	ATGGCAACTCTGATCCCAAC	CGGTCATTGAACTGGTTCCT
DEFB1	TGAGAACTTCCTACCTTCTGCTG	GGTCACTCCCAGCTCACTTG
TRPM8	CAAGTGTTGCTGCAAGGAGA	GAGGTGTCGTTGGCTTTTGT
MMP13	TGCAGCTGTTCACTTTGAGG	TGACGCGAACAATACGGTTA
MMP1	CTACACGGATACCCCAAGGA	AACTTTGTGGCCAATTCCAG
IL1A	CAGTGCTGCTGAAGGAGATG	AACAAGTTTGGATGGGCAAC
CXCL12	AGAGCCAACGTCAAGCATCT	CAGAGCTGGGCTCCTACTGT
ALDH1A1	GCATTGCCAAAGAGGAGATT	CACTTACCACGCCATAGCAA
CFH	TGGAAGATGGGATCCAGAAG	TGAGGTGGTTGTGAACATGG
TUBB1	CGAAGGGATGGACATAAACG	TCCTCCGTGACCTCTTCATC
18S RNA	AACCCGTTGAACCCCATT	CCATCCAATCGGTAGTAGCG

## Results

### Analysis of RUNX2 protein expression and DNA methylation status in serous EOC tumors

Previously, we have identified the RUNX2 gene as hypomethylated and overexpressed in post-CT PCCs, derived from two serous EOC patients, when compared to matched PCCs, obtained prior to CT [9]. Here, we further evaluated RUNX2 protein expression by IHC in serous EOC tumors and ovarian normal tissue samples, using TMAs. Our TMAs included triplicate cores of 117 serous EOC tumors, including 13 LMP tumors, 52 high-grade tumors and 52 omental metastases. Thirteen normal ovarian tissue samples and 13 uterine smooth muscle tissues were also included as controls. Table 1 shows the major clinical characteristics of these patients for whom extensive follow-up clinical data (up to 5-years) were available. The patient's age ranged from 41 to 83 years (median: 64 years). High-grade tumors were mainly grade 3 (99%) and stage III (80%). The majority of patients (87%) received a combination of platinum and paclitaxel. The median baseline CA125 was around 800 U/ml. Forty-three percent of the patients had a progression or a recurrence within the first 6 months of follow-up; for 35.4% of the patients the progression-free survival (PFS) interval was in the range of 7 to 24 months, and 21.2% of the patients displayed PFS values higher than 25 months (see Table S1 for detailed clinicopathological characteristics).

**Figure 1:**
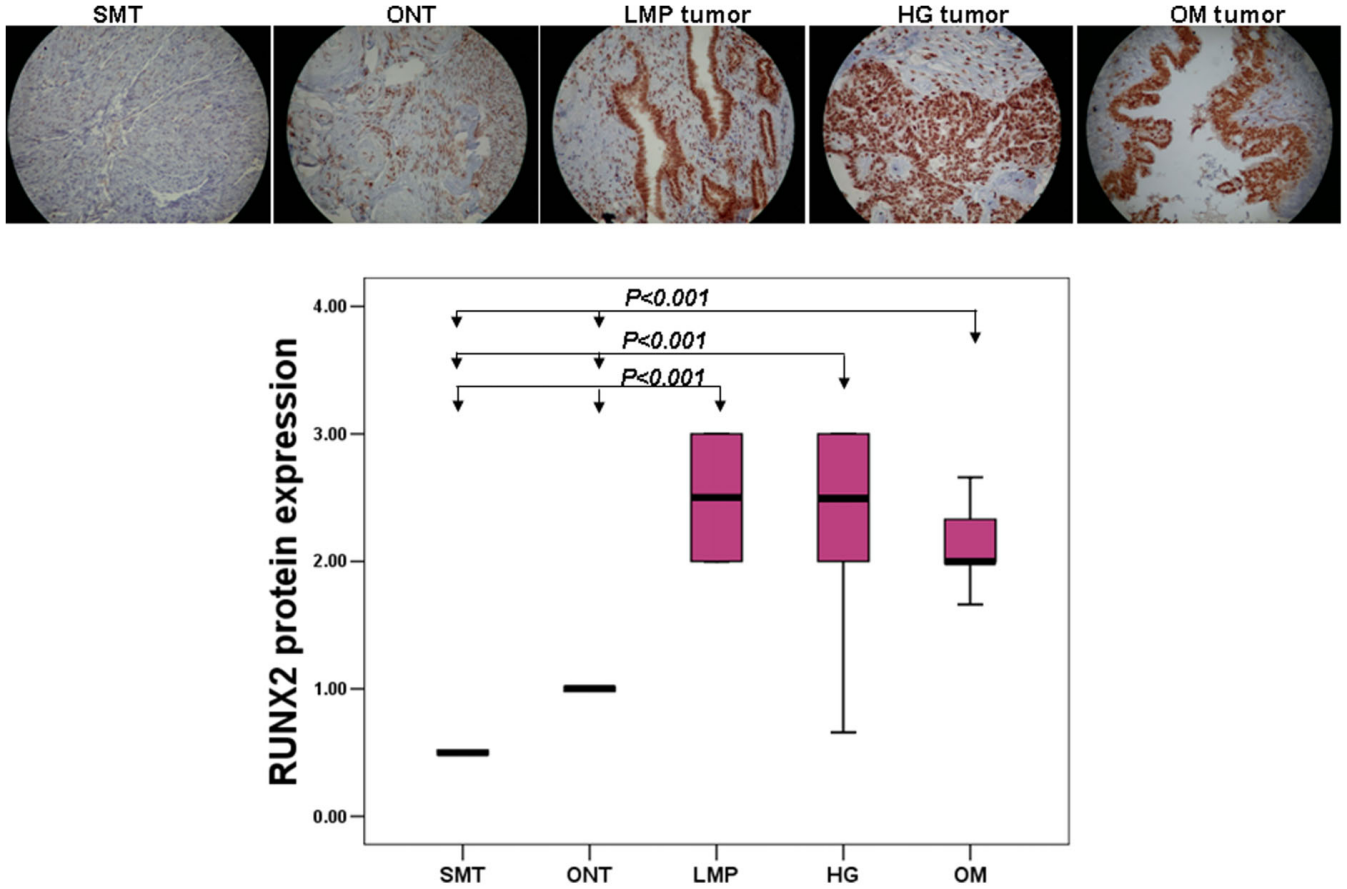
Analysis of RUNX2 expression in serous EOC tumors by IHC. A. Representative IHC images of RUNX2 protein expression in uterine smooth muscle tissues (SMT), normal ovarian tissues (ONT), LMP tumors, high-grade (HG) tumors and omental metastases (OM). B. Box-plot presentation of RUNX2 protein expression levels in SMT, ONT, LMP tumors, HG tumors and OM tumors. See Table S4 for statistical analyses.

Surprisingly, RUNX2 displayed significantly higher expression not only in metastatic tissues (n = 52), but also in 13 LMP and 52 high-grade primary tumors, when compared to 13 normal ovarian tissue samples or 13 normal tissues of other origin (uterine smooth muscle; see Figure 1 and Table S4). Kaplan–Meier survival curves based on RUNX2 expression analyses in cohort of 52 high-grade serous ovarian adenocarcinoma patients displayed no association with PFS (see Figure S1).

**Figure 2:**
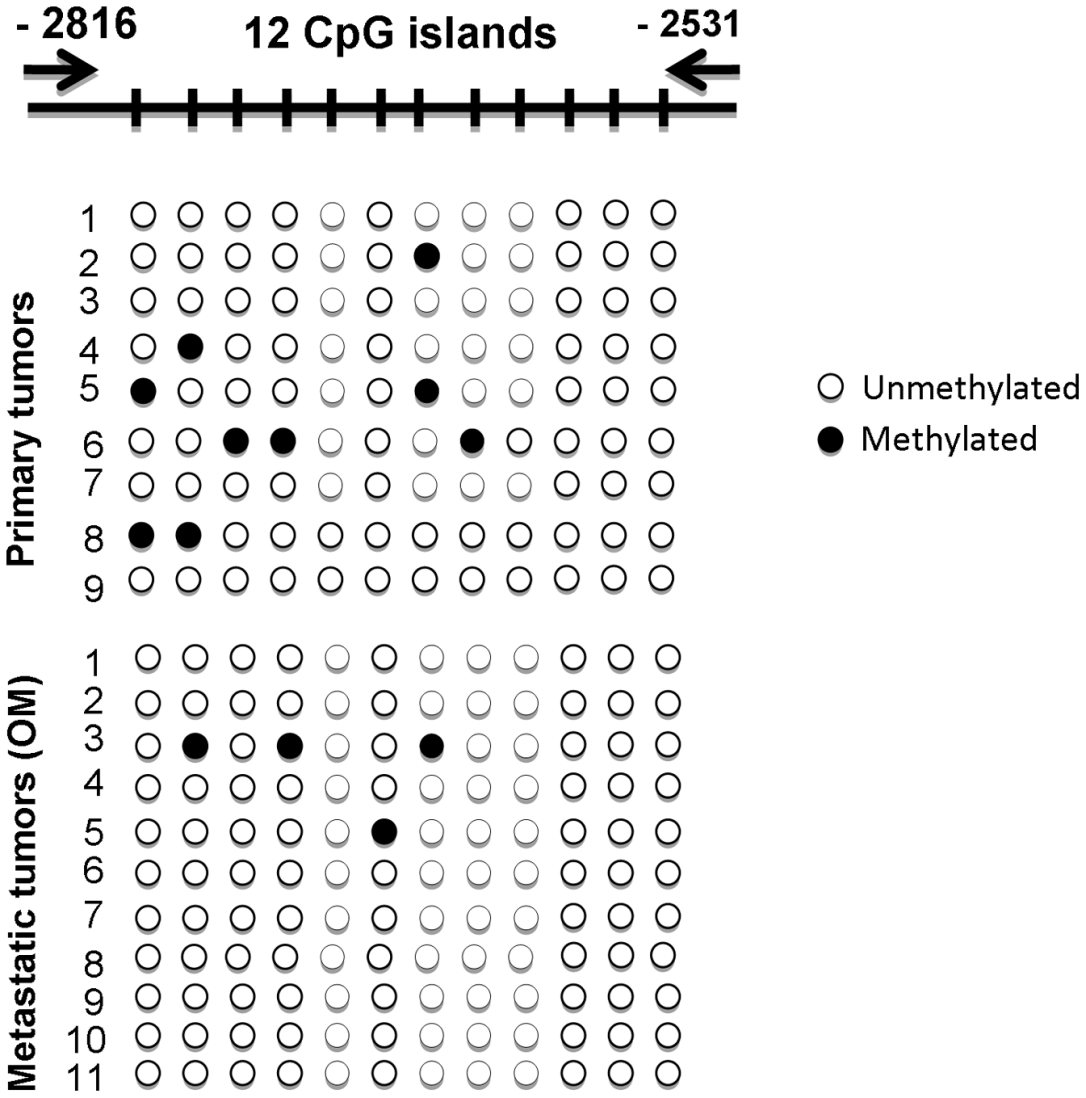
BSP analysis of the methylation status of RUNX2 in grade 3 primary serous EOC tumors compared to omental metastases. Filled circles represent methylated CpGs and open circles represent unmethylated CpGs. CpG plot of the analyzed region is also presented (CpGs are displayed with vertical marks). The indicated positions on the CpG plot represent the number of nucleotides stretching upstream of the first exon of the RUNX2 gene.

We also validated the RUNX2 methylation status in primary tumors and omental metastases. BSP analysis was performed targeting a 285 bp DNA fragment of the proximal promoter (P2) region of RUNX2 gene, stretching between nt −2816 to −2531 upstream of the ATG (start) codon of RUNX2 isoform 3, and containing 12 putative CpG methylation targets (Figure 2; see also Figure S2 for RUNX2 gene structure in relation to these CpG sites). As seen in Figure 2, the BSP analysis displayed no specific RUNX2 hypomethylation in metastatic tissues, compared to primary EOC tumors. These findings suggest that the DNA hypomethylation has no impact on RUNX2 expression in advanced (metastatic) stage of the disease. No differences were also observed when comparing RUNX2 methylation status between primary EOC tumors and normal ovarian tissue samples (see Figure S3).

### Phenotype analysis of RUNX2 suppression in EOC cells: possible implications in EOC cell proliferation, migration and invasion

**Figure 3:**
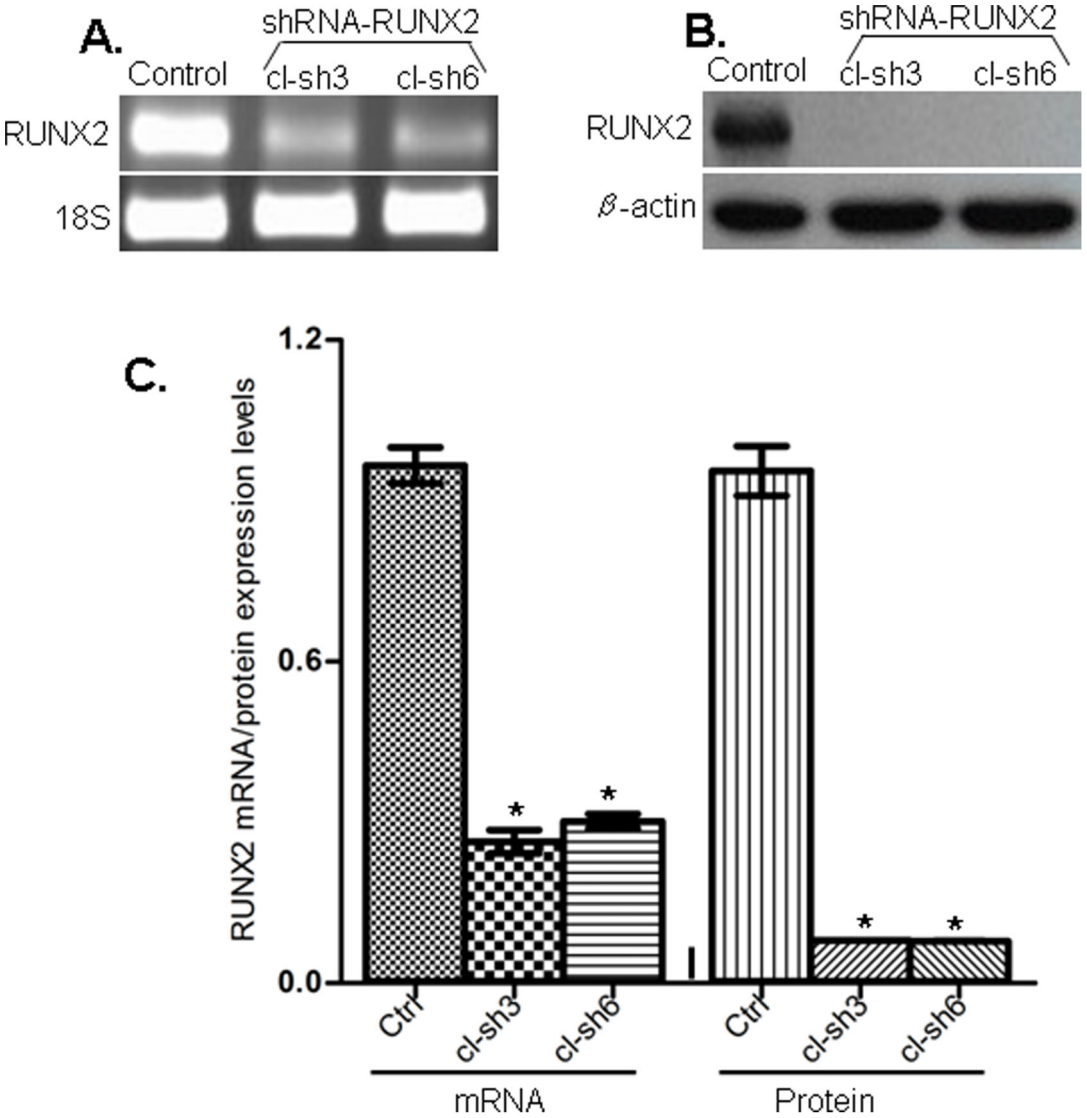
Analysis of RUNX2 expression in SKOV3 cells. A. Semi-quantitative duplex RT-PCR analysis of RUNX2 mRNA expression levels in the shRNA-RUNX2 clones 3 and 6, compared to the mock-transfected control clone. Displayed are images of representative results following sqRT-PCR analysis. The 18S ribosomal RNA gene was used as internal standard. B. Western-blot analysis of RUNX2 protein expression in the shRNA-RUNX2 clones cl-sh3 and cl-sh6, compared to the mock-transfected clone (ctrl). β-actin was used as a loading control. C. Densitometric analysis of RUNX2 mRNA/protein expression levels in the clones sh3 and sh6, compared to the control. Differences between the control clone and shRNA-RUNX2 clones were determined by a Student's t-test. Error bars denote ± SEM; *indicates statistical significance (*P*<0.05).

Next, we verified whether shRNA-mediated RUNX2 gene knockdown could produce any cancer-related phenotypic changes in EOC cells. We tested several EOC cell lines for endogenous RUNX2 expression by sqRT-PCR and Western analysis (see Figure S4). Among these, the SKOV3 and the A2780s cell lines displayed strong RUNX2 expression and were further used to generate stable RUNX2 knockdown clones using the shRNA approach. Clone selection for further analyses was based on sqRT-PCR and Western blot validation of the RUNX2 gene/protein expression in selected clones, compared with non-silencing -transfected clones. Among the clones analyzed, the SKOV3 shRNA-RUNX2 knockdown clones 3 (cl-sh3) and 6 (cl-sh6) and the A2780s clones sh1 and sh2 displayed significant decrease of RUNX2 mRNA and protein expression levels compared to the mock-transfected control (see Figure 3 and Figure S5) and were selected for further analyses.

**Figure 4:**
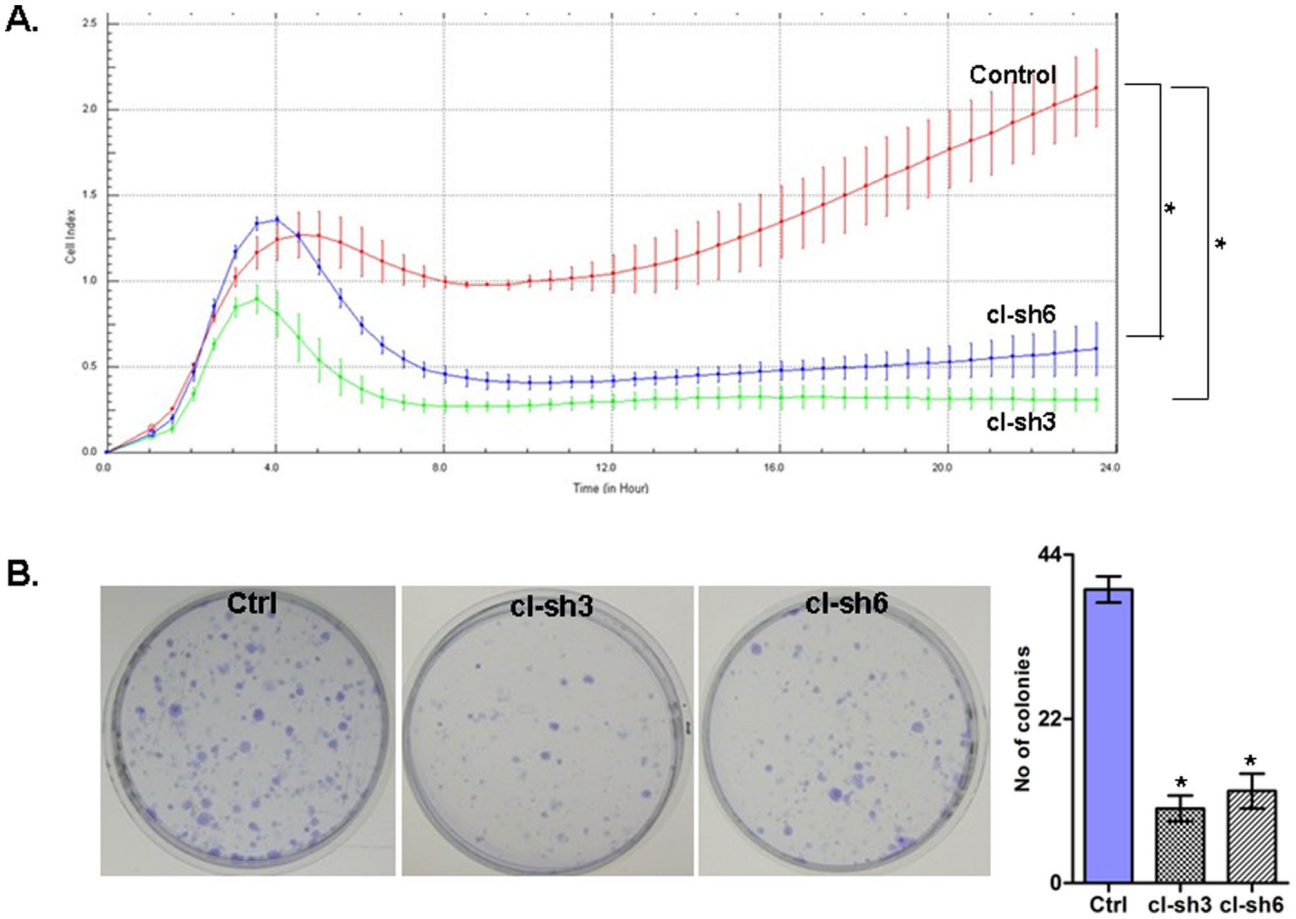
ShRNA-mediated knockdown of the RUNX2 expression in SKOV3 cells. A, effect on cell proliferation; B, Representative images of colony forming assays following RUNX2 knockdown. Error bars denote ± SEM; *indicates statistical significance (*P*<0.05).

**Figure 5:**
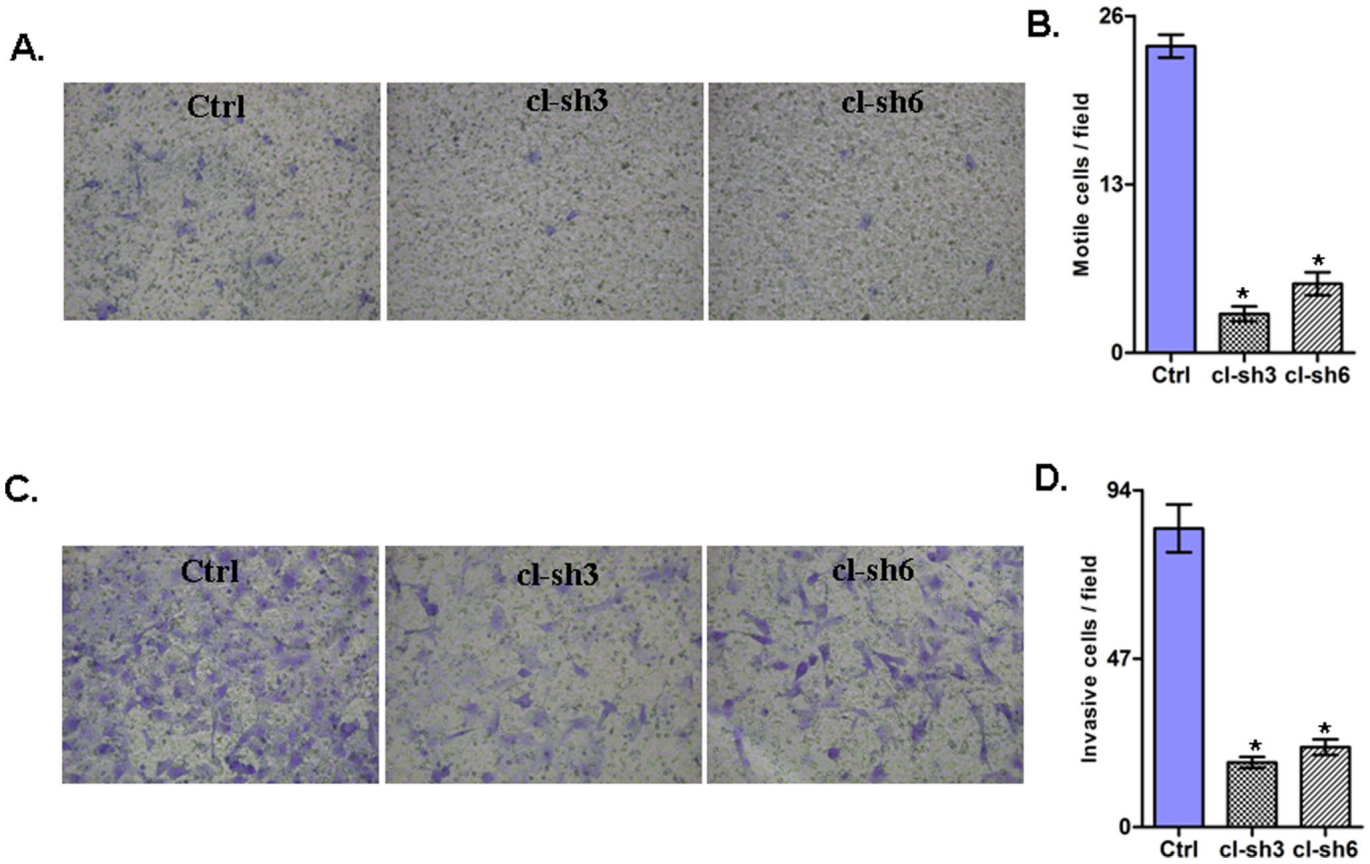
Effect of RUNX2 knockdown on SKOV3 cell migration and invasion. A. Migration was assessed using Boyden-chamber assay. Cells from the shRNA-RUNX clones 3 and 6 and the control clone were seeded into the upper chambers in 0.1% FBS containing medium at a density of 2.5×10^4^ per well, and 600 µl of 1% FBS containing medium was placed in the lower chamber as a chemoattractant. After 24 h at 37°C in 5% CO_2_, the cells were fixed with cold methanol and stained with blue trypan solution. Migrated cells on the underside of the filter were photographed and counted by phase contrast microscopy. B. Cell invasion was assayed in a similar way, as the upper chambers were coated with Matrigel. Here, NIH3T3 conditioned medium was added in the lower chamber as a chemoattractant (see Materials and Methods for details). All experiments were performed in triplicate. For each experiment, cell number was calculated as the total count from 10 random fields per filter (at magnification ×40). Differences between shRNA-RUNX2-transfected and vehicle-transfected SKOV3 cells were determined by a Student's t-test, where *p*<0.05 was considered significant.

We investigated the impact of RUNX2 gene suppression on SKOV3 cell proliferation, cell cycle control, migration, invasion and sensitivity to cisplatin and paclitaxel (drugs, conventionally used for first-line EOC CT). The RUNX2 gene knockdown led to a sharp decrease of the number of viable adherent cells (represented by cell index), compared to control cells (Figure 4A). This observation was further supported by the colony formation assay showing that the numbers of clones formed by cells with stably reduced RUNX2 expression were significantly lower than that of control cells (Figure 4B). Taken together, our observations strongly indicate an influence of RUNX2 transcripts on EOC cell proliferation and further on their propensity to form colonies. Moreover, RUNX2 suppression significantly inhibited both migration and invasion of SKOV3 cells. As shown in Figure 5A and 5B, the numbers of SKOV3 cells that passed through the filter using shRNA clones 3 and 6 were remarkably less than that in the control clone, which is indicative for a role for RUNX2 in the regulation of invasion and migration in EOC. Similar results were obtained upon RUNX2 knockdown in A2780s cells (see Figure S5).

Finally, RUNX2 suppression had no significant impact on SKOV3 cell cycle control and cisplatin and paclitaxel sensitivity (see Figures S6 and S7).

### Molecular mechanisms of RUNX2 action in EOC cells

To better understand the molecular mechanisms of RUNX2 action in EOC cells, we employed the Agilent Whole Human Genome microarrays, containing ∼44,000 genes to identify global gene expression changes upon RUNX2 suppression in SKOV3 cells. We compared the gene expression of the shRNA- RUNX2-clone 3 (cl-sh3) against the corresponding control clone. (Clone 3 was selected for the microarray experiments, since it displayed more profound inhibitory effects on SKOV3 proliferation, migration and invasion, compared to shRNA- RUNX2-clone 6: see Figs. 4 & 5). All microarray experiments were performed in duplicates, as two hybridizations were carried out for the RUNX2-suppressing cell clone against the corresponding control, using a fluorescent dye reversal (dye-swap) technique. For each comparison, a subset of differentially expressed genes was selected displaying at least 2-fold difference in both duplicate microarray experiments. Using these selection criteria, we found 87 genes to be upregulated and 251 genes to be downregulated in SKOV3 cells following RUNX2 knockdown, as the RUNX2 gene displayed 3.36-fold suppression in the shRNA-RUNX2 clone 3 (cl-sh3), compared to the corresponding control (Table S2). Table 3 shows a list of selected functionally related groups of genes that were differentially expressed (≥2-fold) in SKOV3 cells upon RUNX2 knockdown. As seen from Table 3, genes with previously shown implication in mechanisms of metabolism, cell growth & proliferation, regulation of transcription, signal transduction, transport and immune & inflammatory response were predominantly or exclusively suppressed, while RUNX2 knockdown was related with the induction of genes, mostly associated with cell morphology and apoptosis. Table S2 shows the complete list of the differentially expressed genes (≥2-fold) following RUNX2 knockdown in SKOV3 cells.

**Table 3:** Selected differentially expressed gene groups in SKOV3 cells upon RUNX2 knockdown.

A. Upregulated genes	
metabolism	*BC006267, BC035691, CILP, COX7A1, COX7B, DHCR24,. DLST, GALNT14, GNTIVH, PRPS1, SORL1*
signal transduction	*ADAM18, ANXA6, , FBXW11, GPR110, LRRC17, PDAP1, PDZK1IP1, SEPT9, WDR76, WNT6, WNT7A*
cell morphology	*ARHGDIA, EMD, KRT17, LMNB1, LOC201175, MYH6, VIL2, WASF2*
apoptosis	*BCL2L1, CRYAB, CTGF, EZR, JAK1, HFK4, HIST1H1C, ITGB2*
regulation of transcription	*AY517556, EYA4, NEUROG3, ZCCHC2, ZFHX4, ZNF467*
ubiquitination	*BC018548, CGI-301, C15orf16, RFWD3, RKHD1, TTC3*

B. Downregulated genes	
metabolism	*ACAT1, ACOX2, AK096526, ALDH1A1, ANGPTL4, ASNS, B3GALT3, BPNT1, CARS, CPE, CTH, DDX50, DHRS2, DMGDH, HSD17B12, ISOC1, KYNU, LOC440138, MARS, MLSTD2, MMP1, MMP13, MMP19, MTHFD2, NAALADL1, PCK2, PHGDH, PPP1CB, PSAT1, PTGS1, RBP1, SAT, SENP7, SERPINA5, SHMT2, SORD*
cell growth & proliferation	*ADRA2A, BTG1, CLK1, CNN1, COVA1, DDIT3, EPS8, FGF2, FRMD4A, G0S2, GADD45A, GDF15, HIST1H1A, IL1A, JEM-1, KIF3B, MORF4L2, P8, POLI, POPDC2, PRKRIR, RPS6KA5, SEPT10, SIAH1, TUBB1*
regulation of transcription	*AF024709, ATF1, ATF4, BX538289, CMPX1, ETV5, HEY1, HLF, LOC340501, NFE2, PHLDA1, RCOR3, RUNX2, TBX15, TSC22D1, ZFP90, ZNF140, ZNF146, ZNF22, ZNF354C, ZNF396, ZNF441, ZNF451, ZNF639*
signal transduction	*ANTXR2, CNIH3, DSCR1, ECT2, GRINL1A, IL13RA2, MAPK9, MRFAP1, MRFAP1L1, PRKAR1A, PTGES3, RAGE, RASSF6, STC2, STMN3, TANK, TRIB3, TXNIP, TXNRD1, UNC5B*
transport	*FLJ35848, HRC11236, KCNE3, RBP4, SLC40A1, SLC9A9, STEAP1, STX6, TARS, TOM1L1, TRPA1, TRPM8, VPS13B*
immune & inflammatory response	*CD48, CEBPB, CFH, , CFH, CFHL1, CFHL3, CXCL12, IF, MIG-6, NFIL3, PRKRA, PTX3, SELE, SEPP1*
ubiquitination	*FBXO32, FBXO4, FTS, HERC3, LOC51136, RKHD2, RNF34, RNF6, TRIM4, TRIM40*

**Figure 6:**
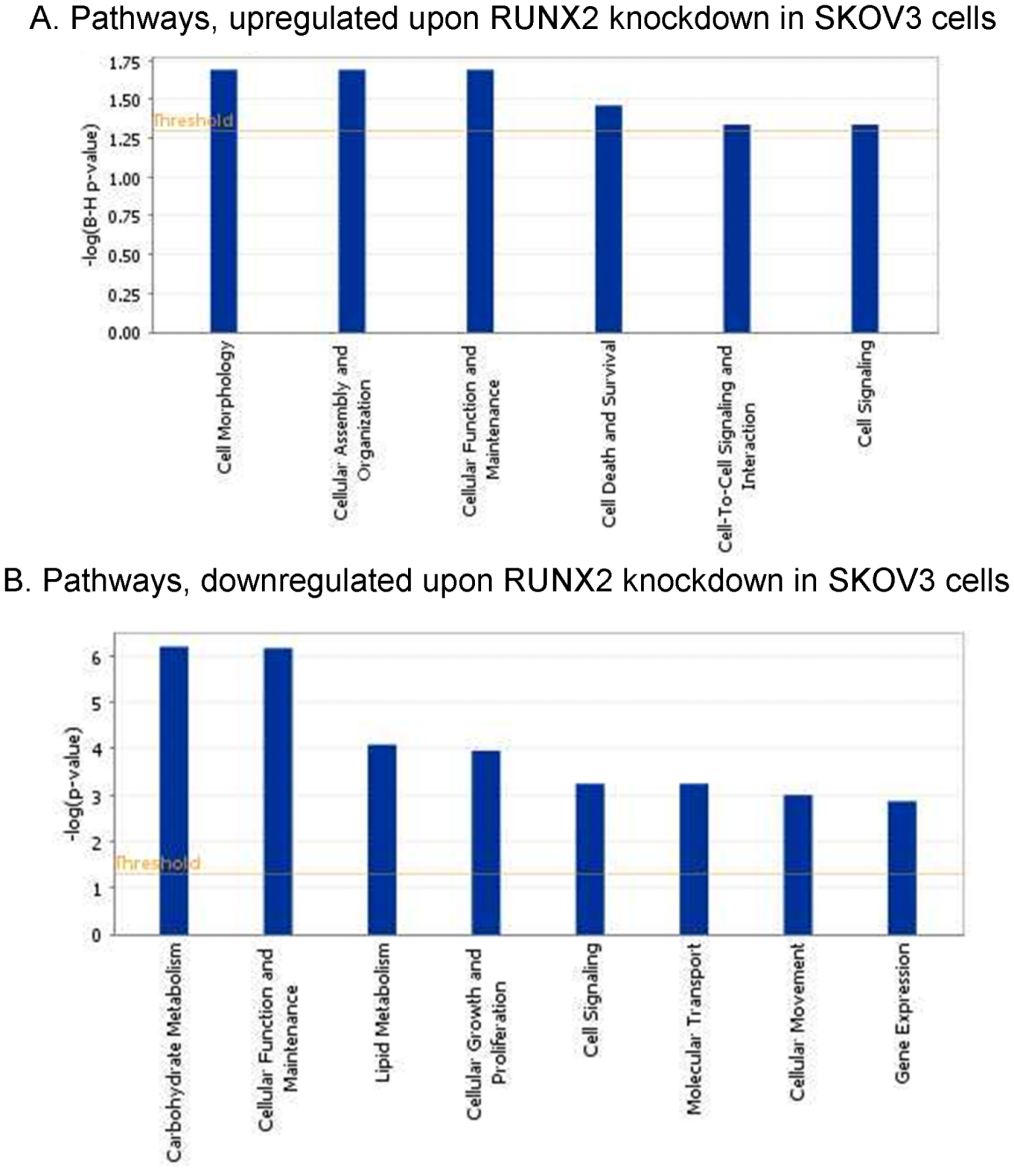
Functional analysis for a dataset of differentially expressed genes (≥2-fold) following RUNX2 suppression in SKOV3 cells. A. Functional analysis of upregulated genes; B. Functional analysis of downregulated genes. Top functions that meet a *p*-value cutoff of 0.05 are displayed.

Pathway and network analyses, generated through the use of the IPA software confirmed the major functionally related gene groups, found to be differentially expressed in the shRNA-RUNX2-cl3 clone. As seen from Figure 6, pathways implicated in cellular morphology, cell death and survival and cell-to-cell signaling and interaction were predominantly upregulated (Figure 6A), while pathways linked to carbohydrate and lipid metabolism, cell growth and proliferation, molecular transport, cellular movement and gene expression were mostly suppressed (Figure 6B).

**Figure 7:**
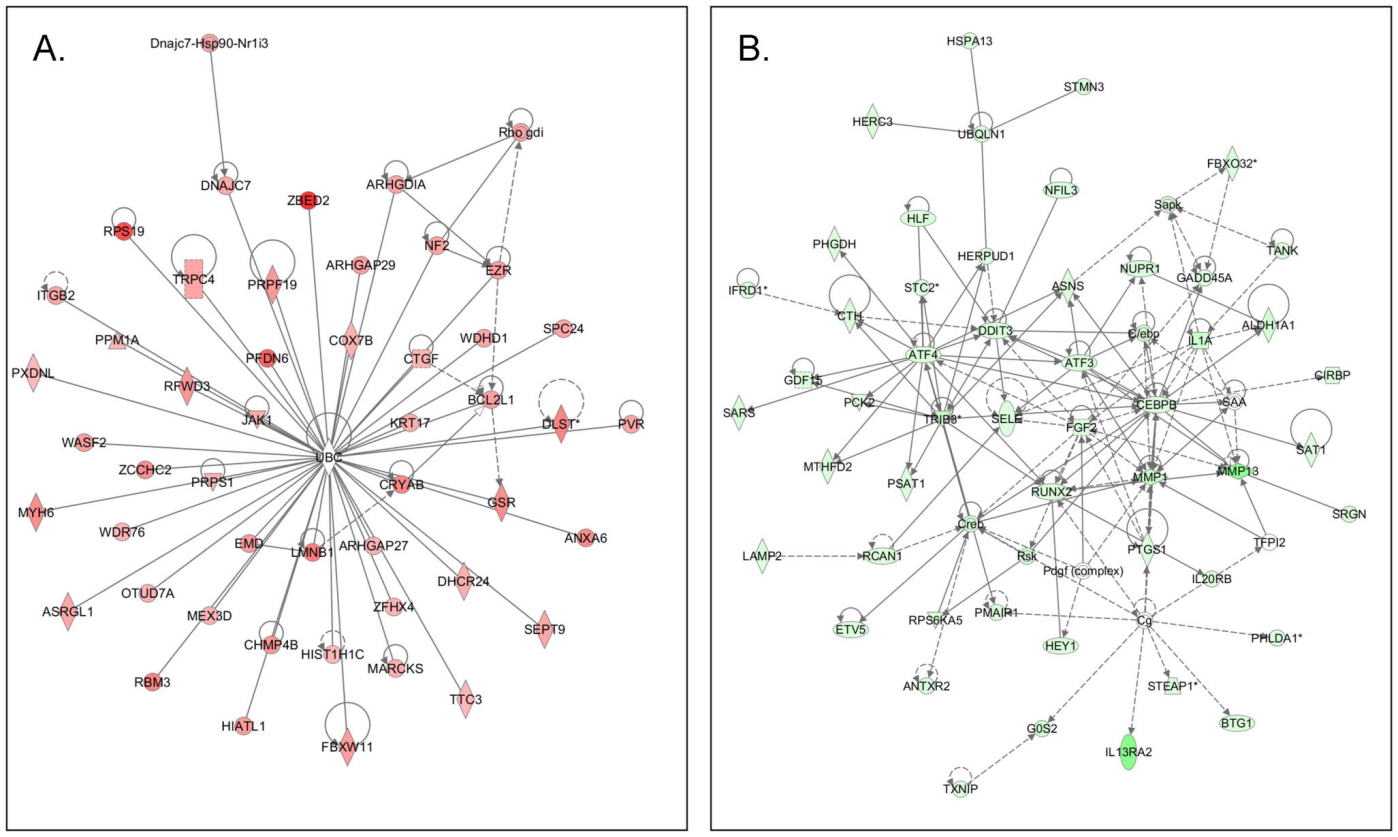
Network analysis of dynamic gene expression in SKOV3 cells based on the 2-fold common gene expression list obtained following shRNA-mediated RUNX2 knockdown. A. Upregulated networks; B. Downregulated networks. The five top-scoring networks for each cell line were merged and are displayed graphically as nodes (genes/gene products) and edges (the biological relationships between the nodes). Intensity of the node color indicates the degree of up- (red) or downregulation (green). Nodes are displayed using various shapes that represent the functional class of the gene product (square, cytokine, vertical oval, transmembrane receptor, rectangle, nuclear receptor, diamond, enzyme, rhomboid, transporter, hexagon, translation factor, horizontal oval, transcription factor, circle, other). Edges are displayed with various labels that describe the nature of relationship between the nodes: ^____^ binding only, → acts on. The length of an edge reflects the evidence supporting that node-to-node relationship, in that edges supported by article from literature are shorter. Dotted edges represent indirect interaction.

Common networks obtained upon merging the top-scoring networks recognized some important gene nodes and genes that are specifically up- or downregulated upon RUNX2 suppression in SKOV3 cells (Figure 7). Thus, genes and associated interaction partners that were upregulated upon RUNX2 knockdown (displayed on Figure 7A) comprised members of the ubiquitin C (UBC) interaction network, including genes, predominantly implicated in cell morphology (KRT17, LMNB1, MARCKS, MYH6, PVR, SEPT9, WASF2) and apoptosis (ARHGDIA, BCL2L1, CRYAB, CTFG, EZR, ITGB2). Major gene nodes that were downregulated upon RUNX2 knockdown in SKOV3 cells are presented in Figure 7B; these were mostly involved in metabolism (MMP1, MMP13, PTGS1/COX1/COX1), cell growth & proliferation (GADD45A, DDIT3, FGF2, IL1A), regulation of transcription (ATF3, ATF4, RUNX2), signal transduction (Creb, TRIB3, Sapk), immune & inflammatory response (CEBPB, C/ebp, SELE) and ubiquitination (UBQLN1).

### Validation of microarray findings with quantitative PCR (qPCR)

**Figure 8:**
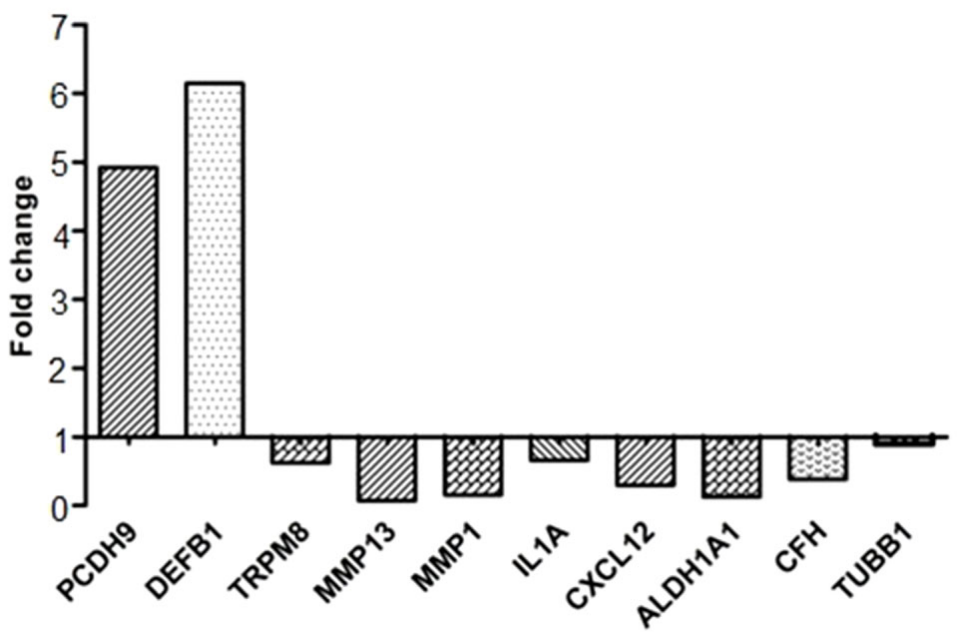
Quantitative PCR validation of microarray results. The figure shows bar graphs presentation of the differential expression of the selected genes in the shRNA-RUNX2 clone 3 (cl-sh3) compared to the control clone. The relative copy number was calculated based on the target gene/18S ribosomal RNA ratio. Values more than or equal to 1 represent gene upregulation and more than 1 display gene downregulation. The analysis confirmed a higher level of PCDH9, DEFB1 expression and lower levels of TRPM8, MMP13, MMP1, IL1A, CXCL12, ALDH1A1, CFH, TUBB1 expressions in the cl-sh3 clone.

To validate microarray results, we arbitrarily selected 10 differentially expressed genes and quantified their expression by qPCR in SKOV3 cells following shRNA-RUNX2 knockdown compared to control (vehicle transfected) SKOV3 cells. Figure 8 summarizes the gene expression measurements of all validated genes. We found that both methods (microarray analysis and qPCR) detected similar patterns for the up- and down-regulated genes selected for validation.

### RUNX1 and RUNX2 use distinct molecular mechanisms to promote ovarian cancer cell proliferation, migration and invasion

**Figure 9:**
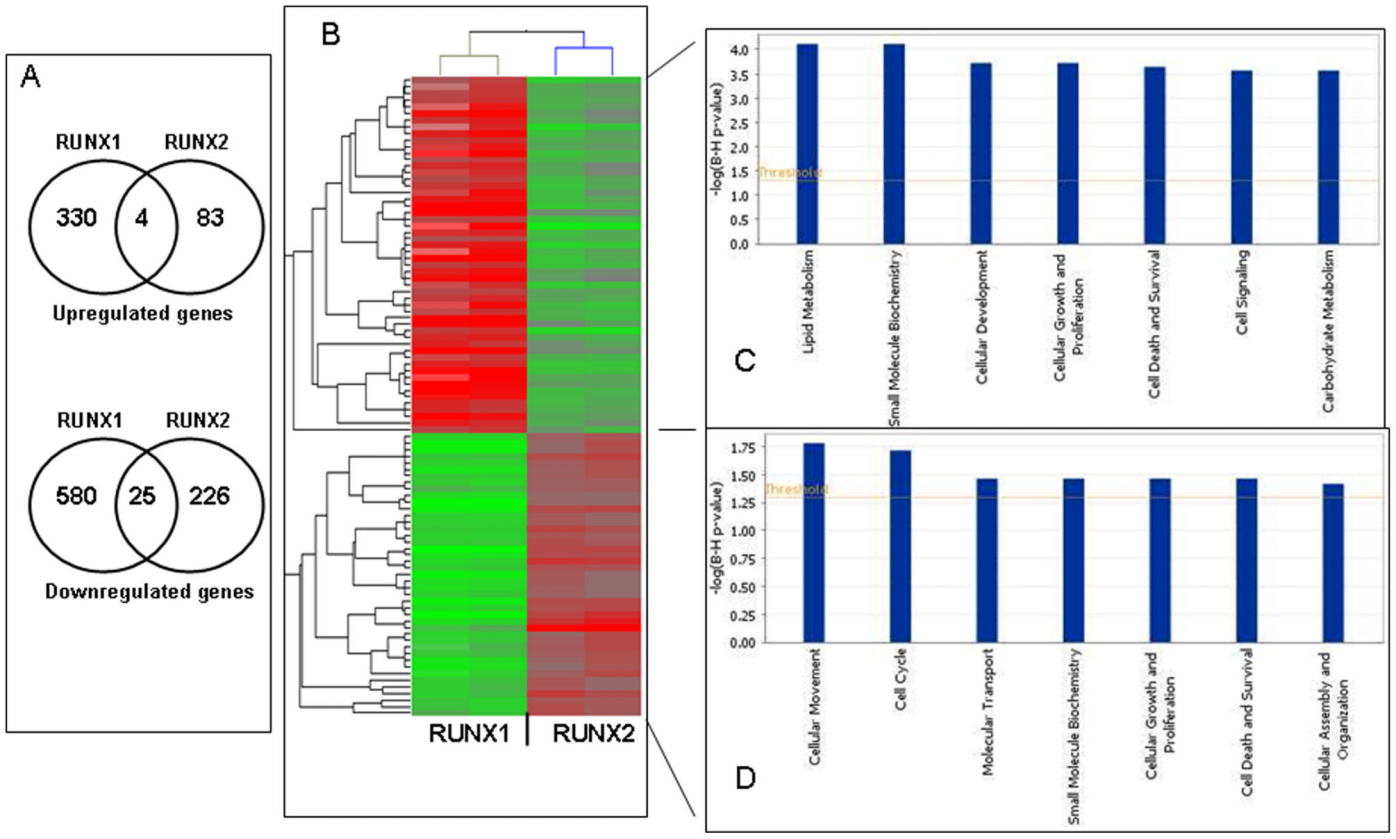
Comparison of common and distinct gene expressions across the various differentially-expressed gene groups upon RUNX1 and RUNX2 knockdown in SKOV3 cells. A. Venn diagram analyses of the differentially expressed genes upon RUNX1 and RUNX2 knockdown in SKOV3 cells. B. Hierarchical clustering based on the 95-genes list (2-fold difference in gene expression; p-value cutoff of 0.05) that discriminates differentially-expressed genes in SKOV3 cells upon RUNX1 and RUNX2 knockdown. Red signifies up-regulation, and green signifies down-regulation. C. IPA functional pathway analyses of genes, differentially expressed in SKOV3 cells upon RUNX1 and RUNX2 knockdown, based on the 95-genes list generated upon the clustering analysis.

Our functional analyses were strongly indicative for similar roles of RUNX1 and RUNX2 in EOC progression, including implication in EOC cell proliferation, migration and invasion (see [25] and the data above). This prompted us to compare the microarray data obtained upon RUNX1 and RUNX2 knockdown in SKOV3 cells in order to get insight of the specific and/or common mechanisms of RUNX1/RUNX2 action in EOC. Venn diagram comparison analyses were indicative for negligible number of commonly overexpressed or suppressed genes following RUNX1 and RUNX2 suppression (Figure 9A). This was further confirmed by clustering analysis, as following filtering on 2-fold signal intensity, we used one-way ANOVA parametric test (Welch *t*-test; variances not assumed equal) to select discriminatory genes. Indeed, *t* test with *p*-value cutoff of 0.05 selected 95 genes for which expression differed in shRNA-RUNX1 SKOV3 cell clones compared to shRNA-RUNX2 SKOV3 cell clones. Clustering analysis based on the 95-genes list was performed using the standard Condition Tree algorithm provided in GeneSpring, and revealed formation of two major cluster groups that clearly distinguish SKOV3 cells upon RUNX1 and RUNX2 knockdown (Figure 9B). Fifty two genes from the 95-genes list were relatively downregulated in SKOV3 cells upon RUNX2 knockdown compared to RUNX1 knockdown, as major functional classifications of these genes predominantly include metabolism, cellular development and cell signaling (Figure 9C). Genes, mostly downregulated in shRNA-RUNX1 clones when compared to shRNA-RUNX2 cell clones were mainly involved in cellular movement, cell cycle control and molecular transport (Figure 9D). The 95-genes list is presented in Table S3.

However, we cannot completely exclude some common mechanisms of RUNX1/RUNX2 action in EOC cells, since both RUNX1 and RUNX2 knockdown leads to the suppression of the pro-metastatic gene p8/NUPR1 [40], [41], as well as the downregulation of genes (MMP1, MMP19, PTGS1/COX1) with proven role in EOC progression and dissemination [42]–[44].

## Discussion

RUNX proteins have been demonstrated to play positive and negative roles in carcinogenesis according to different cancer types [11]. The RUNX2 gene, also known as CBF, runt domain, a-subunit 1, CBFA1, AML3, or OSF2, is a lineage-specific transcription factor and the human homolog of mouse PEBP2A [45]. During embryonic development, RUNX2 is involved in the process of bone formation or osteogenesis [14]. In carcinogenesis, RUNX2 acts as a master regulator of disease progression, and was shown to be strongly implicated in the development of osteosarcoma [46]. In addition, overexpression of RUNX2 has also been identified in several human malignancies, including lymphoma/leukemia [26], bone [27], breast [28], prostate [29], thyroid [30] and pancreatic cancers [31]. Similarly, overexpression of RUNX2 has been reported in EOC tissues compared with normal ovarian tissues, and its upregulation was closely related with the clinical stage and poor prognosis of EOC patients [32]. In most of these studies, RUNX2 was functionally associated with tumor invasion and metastasis. Indeed, Akech et al. [29] indicated that RUNX2 is associated with prostate cancer bone metastasis and maybe a potential therapeutic target to block prostate cancer cells ability for tumor growth and metastatic lesions formation *in vivo*. The results of Niu et al. [30] group revealed that RUNX2 is functionally linked to tumor invasion and metastasis of thyroid carcinoma by regulating EMT-related molecules, matrix metalloproteinases and angiogenic factors. Pratap et al. [28] found that RUNX2 expression may play important role in breast tumor cell invasion. A recent study identified RUNX2 as a potent prognostic factor in non-small cell lung cancer (NSCLC) patients, as RUNX2 expression was significantly correlated with NSCLC tumor progression and metastatic capability [47].

Our previous findings based on analyses in PCCs derived from matched tumor samples obtained prior to, and following CT treatment from two serous EOC patients, were suggestive for RUNX2 overexpression in advanced (metastatic) EOC, which might be due to epigenetic mechanisms, associated with DNA hypomethylation of its putative promoter region [9]. However, in the present study we show that the CpG island located in this promoter region displays no significant hypomethylation in EOC omental metastases, compared to primary serous EOC tumors. Thus, our data point to no implication of epigenetics mechanisms (DNA hypomethylation) in RUNX2 overexpression in metastatic tissues. Thus, it is highly probable that the previously observed DNA methylation differences in post-CT PCCs, when compared to pre-CT PCCs, are due to the CT treatment. Moreover, our IHC analyses were indicative for strong RUNX2 protein overexpression both in grade 3 serous EOC tumors and metastatic tissues. Interestingly, RUNX2 also displayed significantly increased expression in LMP tumors, compared to normal ovarian tissue. Normal ovarian tissue controls consistently displayed low RUNX2 expression; minimal expression was also detected in other human adult tissues (uterine smooth muscle).

The above findings persuaded us to investigate the functional implication of RUNX2 in mechanisms of EOC tumorigenesis. Our functional analyses are strongly indicative for evident oncogenic capacity of RUNX2 in serous EOC, including its potential role in EOC cell proliferation and cell migration/invasion (see Figs. 4, 5 and Fig. S5). Thus, our data confirm recent findings [28]–[31] suggesting that RUNX2 promotes tumor and cancer cell growth and/or invasion/metastasis.

To better elucidate the molecular mechanisms and biological pathways implicated in RUNX2-mediated action in EOC cells, we used a complementary gene expression profiling using the DNA microarray technology to monitor cellular changes in gene expression and discover the molecular targets upon RUNX2 suppression in EOC cells. To our knowledge, the present work represents the first effort to define global changes in gene expression upon modulation of RUNX2 expression in cancer cells. We analyzed both functionally related genes that were commonly differentially expressed in SKOV3 EOC cells upon RUNX2 knockdown. The gene expression data and consecutive network and pathway analyses were quite confirmatory of the data obtained by the RUNX2 functional assays. Indeed, microarray data sustained RUNX1 correlation with EOC cell proliferation, migration and invasion, since RUNX2 knockdown resulted in reduced expression of genes associated with metabolism, cellular growth & proliferation and cellular movement, while a number of genes linked to cell death were induced (see Table 3 and Figure 6).

IPA network analysis was indicative for some important gene nodes linked to RUNX2 suppression in EOC cells, as most of these substantiate and/or complement the functional data obtained. Thus, RUNX2 knockdown resulted in upregulation of gene nodes/genes known to be implicated in apoptosis induction or displaying TSG functions (see Figure 7A). Notably the UBC interaction network and its members were shown to decrease in anchorage-independent cell growth and increase apoptosis, suggesting UBC may act as a negative regulator of skin carcinogenesis [48]; CRYAB has been reported as a potential TSG [49], while increased expression of BCL-XS (BCL2L1) protein in tumors was associated with decreased proliferation and induction of apoptosis [50], [51]. Similarly, CTGF upregulation was found to be associated with apoptosis and decrease of tumor cell invasion [52]–[54]; PPM1A (PP2C) expression could induce cell cycle arrest and apoptosis via activation of the p53 pathway [55], and NF2 has been characterized as a TSG in different cancers [56]–[58].

In parallel, upon RUNX2 knockdown, we have observed a predominant and strong downregulation of gene nodes known to be implicated in EOC tumorigenesis (PTGS1/COX1, FGF2, IL1A, Sapk, C/ebp, SELE, UBQLN1, PSAT1, ALDH1A1, GDF15, MTHFD2) [59]–[68], including EOC tumor invasion/metastasis (MMP1, MMP13, Creb) [69]–[71]; (see Figure 7B). Some of these gene nodes have been shown to be functionally involved in other cancer types, including regulation of tumor cell proliferation (Creb, C/ebp, ATF3, ATF4) [72]–[75], invasion (MTHFD2, FGF2) [76], [77] and metastasis (PTGS1/COX1, E-selectin, ATF3, FGF2, IL1A, MMP1, MMP13) [78]–[83]. Thus, our data support the concept of oncogenic role of RUNX2 in EOC, and support previous findings for its rather universal functions in tumorigenesis, including tumor invasion and metastasis.

Given the similar roles of RUNX1 and RUNX2 in EOC progression (implications in EOC cell proliferation, migration and invasion) and the fact that all three RUNX proteins recognize common DNA sequence motifs [10], we analyzed the extent of overlap in differentially expressed genes/functional pathways following RUNX1 and RUNX2 knockdown in the SKOV3 ovarian adenocarcinoma cell line (analyses based on the data presented herein and our previous findings [25]). Both the Venn diagram comparisons, as well as gene clustering and IPA functional analyses were indicative for distinct molecular mechanisms and functional pathways associated with RUNX1 or RUNX2 implication in EOC progression (see Figure 9), although both genes could potentially modulate the expression of some common genes involved in EOC disease advancement and metastasis (including MMP1, MMP19 and PTGS1/COX1, and possibly p8/NUPR1).

In conclusion, we have shown that the RUNX2 transcription factor is significantly overexpressed in serous EOC tumors, including LMP tumors, compared to normal ovarian tissue. BSP validation of the RUNX2 methylation status in primary EOC tumors and omental metastasis were indicative for no implication of epigenetics mechanisms (DNA hypomethylation) in RUNX2 overexpression in metastatic tissues. Further functional analyses of RUNX2 in EOC cells pointed towards its association with EOC cell proliferation, migration and invasion. Gene expression profiling and consecutive network and pathway analyses confirmed these findings, as various genes and pathways known previously to be implicated in ovarian tumorigenesis, including EOC tumor invasion and metastasis, were found to be suppressed upon RUNX2 knockdown, while a number of pro-apoptotic genes and some EOC TSGs were found to be induced. Our data suggest that RUNX2 is possibly implicated in EOC tumor and cancer cell growth and invasion and could represent a potential EOC therapeutic target. The present study also reveals that RUNX1 and RUNX2 employ distinct molecular mechanisms in EOC tumorigenesis despite evident similarities of their action on EOC cell phenotype and behavior. Taken together, our data are indicative of strong oncogenic potential of both transcription factors in EOC progression and warrant further and more profound studies of the functional implications of the RUNX transcription factors in EOC tumorigenesis.

## Supporting Information

Figure S1 **Kaplan-Meier curve for progression free survival according to the level of RUNX2 IHC intensity in tumor samples of 52 serous EOC patients.**
(PPT)

Figure S2 **Genomic structure of the RUNX2 gene, isoforms a, b and c.** The CpG island (CpG 60) containing the analyzed 12 putative CpG methylation targets is indicated with arrow.
(PPT)

Figure S3 **BSP analysis of the methylation status of RUNX2 in grade 3 primary serous EOC tumors compared to omental metastases.**
(PPT)

Figure S4 **Western blot analysis of RUNX2 protein expression in different EOC cell lines.**
(PPT)

Figure S5 **ShRNA-mediated knockdown of the RUNX2 expression in A2780s cells and consecutive analyses of functional phenotypes.**
(PPT)

Figure S6 **ShRNA-mediated knockdown of the RUNX2 expression in SKOV3 cells: effect on cell cycle control.**
(PPT)

Figure S7 Dose-response cytotoxicity curves upon cisplatin (A) and paclitaxel (B) treatment of SKOV3 cells following shRNA-mediated RUNX2 knockdown.
(PPT)

Table S1 **Patients' clinical characteristics.**
(XLS)

Table S2 **Genes, differentially expressed in SKOV3 cells (≥2 fold) following RUNX2 knockdown.**
(XLS)

Table S3 **List of 95 genes used for cluster analysis.**
(XLS)

Table S4 **Median comparison analysis (Mann-Whitney) of the RUNX2 expression values in different EOC and control tissues.**
(DOC)

## References

[1] Siegel R, Ward E, Brawley O, Jemal A (2011) Cancer statistics, 2011: the impact of eliminating socioeconomic and racial disparities on premature cancer deaths. CA Cancer J Clin 61: 212–236. 21685461 10.3322/caac.20121

[2] Barnholtz-Sloan JS, Schwartz AG, Qureshi F, Jacques S, Malone J, et al (2003) Ovarian cancer: changes in patterns at diagnosis and relative survival over the last three decades. Am J Obstet Gynecol 189: 1120–1127. 14586365 10.1067/s0002-9378(03)00579-9

[3] Alouini S (2012) Management of ovarian cancer has changed. Gynecol Oncol 126: 313; author reply 314. 22561040 10.1016/j.ygyno.2012.04.044

[4] Agarwal R, Kaye SB (2003) Ovarian cancer: strategies for overcoming resistance to chemotherapy. Nat Rev Cancer 3: 502–516. 12835670 10.1038/nrc1123

[5] Jones PA, Baylin SB (2007) The epigenomics of cancer. Cell 128: 683–692. 17320506 10.1016/j.cell.2007.01.029 PMC3894624

[6] Balch C, Fang F, Matei DE, Huang TH, Nephew KP (2009) Minireview: epigenetic changes in ovarian cancer. Endocrinology 150: 4003–4011. 19574400 10.1210/en.2009-0404 PMC2736079

[7] Momparler RL (2003) Cancer epigenetics. Oncogene 22: 6479–6483. 14528271 10.1038/sj.onc.1206774

[8] Maier S, Dahlstroem C, Haefliger C, Plum A, Piepenbrock C (2005) Identifying DNA methylation biomarkers of cancer drug response. Am J Pharmacogenomics 5: 223–232. 16078859 10.2165/00129785-200505040-00003

[9] Keita M, Wang ZQ, Pelletier JF, Bachvarova M, Plante M, et al (2013) Global methylation profiling in serous ovarian cancer is indicative for distinct aberrant DNA methylation signatures associated with tumor aggressiveness and disease progression. Gynecol Oncol 128: 356–363. 23219462 10.1016/j.ygyno.2012.11.036

[10] Huang X, Peng JW, Speck NA, Bushweller JH (1999) Solution structure of core binding factor beta and map of the CBF alpha binding site. Nat Struct Biol 6: 624–627. 10404216 10.1038/10670

[11] Wang CQ, Jacob B, Nah GS, Osato M (2010) Runx family genes, niche, and stem cell quiescence. Blood Cells Mol Dis 44: 275–286. 20144877 10.1016/j.bcmd.2010.01.006

[12] Okuda T, van Deursen J, Hiebert SW, Grosveld G, Downing JR (1996) AML1, the target of multiple chromosomal translocations in human leukemia, is essential for normal fetal liver hematopoiesis. Cell 84: 321–330. 8565077 10.1016/s0092-8674(00)80986-1

[13] Wang Q, Stacy T, Binder M, Marin-Padilla M, Sharpe AH, et al (1996) Disruption of the Cbfa2 gene causes necrosis and hemorrhaging in the central nervous system and blocks definitive hematopoiesis. Proc Natl Acad Sci U S A 93: 3444–3449. 8622955 10.1073/pnas.93.8.3444 PMC39628

[14] Otto F, Thornell AP, Crompton T, Denzel A, Gilmour KC, et al (1997) Cbfa1, a candidate gene for cleidocranial dysplasia syndrome, is essential for osteoblast differentiation and bone development. Cell 89: 765–771. 9182764 10.1016/s0092-8674(00)80259-7

[15] Levanon D, Bettoun D, Harris-Cerruti C, Woolf E, Negreanu V, et al (2002) The Runx3 transcription factor regulates development and survival of TrkC dorsal root ganglia neurons. EMBO J 21: 3454–3463. 12093746 10.1093/emboj/cdf370 PMC125397

[16] Fainaru O, Woolf E, Lotem J, Yarmus M, Brenner O, et al (2004) Runx3 regulates mouse TGF-beta-mediated dendritic cell function and its absence results in airway inflammation. EMBO J 23: 969–979. 14765120 10.1038/sj.emboj.7600085 PMC380997

[17] Blyth K, Cameron ER, Neil JC (2005) The RUNX genes: gain or loss of function in cancer. Nat Rev Cancer 5: 376–387. 15864279 10.1038/nrc1607

[18] Subramaniam MM, Chan JY, Yeoh KG, Quek T, Ito K, et al (2009) Molecular pathology of RUNX3 in human carcinogenesis. Biochim Biophys Acta 1796: 315–331. 19682550 10.1016/j.bbcan.2009.07.004

[19] Zhang S, Wei L, Zhang A, Zhang L, Yu H (2009) RUNX3 gene methylation in epithelial ovarian cancer tissues and ovarian cancer cell lines. OMICS 13: 307–311. 19645591 10.1089/omi.2009.0030

[20] Lee CW, Chuang LS, Kimura S, Lai SK, Ong CW, et al (2011) RUNX3 functions as an oncogene in ovarian cancer. Gynecol Oncol 122: 410–417. 21612813 10.1016/j.ygyno.2011.04.044

[21] Scheitz CJ, Tumbar T (2012) New insights into the role of Runx1 in epithelial stem cell biology and pathology. J Cell Biochem 10.1002/jcb.24453 PMC5788165 23150456

[22] Wotton S, Stewart M, Blyth K, Vaillant F, Kilbey A, et al (2002) Proviral insertion indicates a dominant oncogenic role for Runx1/AML-1 in T-cell lymphoma. Cancer Res 62: 7181–7185. 12499254

[23] Robinson HM, Broadfield ZJ, Cheung KL, Harewood L, Harris RL, et al (2003) Amplification of AML1 in acute lymphoblastic leukemia is associated with a poor outcome. Leukemia 17: 2249–2250. 14523475 10.1038/sj.leu.2403140

[24] Planaguma J, Liljestrom M, Alameda F, Butzow R, Virtanen I, et al (2011) Matrix metalloproteinase-2 and matrix metalloproteinase-9 codistribute with transcription factors RUNX1/AML1 and ETV5/ERM at the invasive front of endometrial and ovarian carcinoma. Hum Pathol 42: 57–67. 20970160 10.1016/j.humpath.2010.01.025

[25] Keita M, Bachvarova M, Morin C, Plante M, Gregoire J, et al (2013) The RUNX1 transcription factor is expressed in serous epithelial ovarian carcinoma and contributes to cell proliferation, migration and invasion. Cell Cycle 12: 972–986. 23442798 10.4161/cc.23963 PMC3637356

[26] Blyth K, Vaillant F, Jenkins A, McDonald L, Pringle MA, et al (2010) Runx2 in normal tissues and cancer cells: A developing story. Blood Cells Mol Dis 45: 117–123. 20580290 10.1016/j.bcmd.2010.05.007

[27] Martin JW, Zielenska M, Stein GS, van Wijnen AJ, Squire JA (2011) The Role of RUNX2 in Osteosarcoma Oncogenesis. Sarcoma 2011: 282745. 21197465 10.1155/2011/282745 PMC3005824

[28] Pratap J, Javed A, Languino LR, van Wijnen AJ, Stein JL, et al (2005) The Runx2 osteogenic transcription factor regulates matrix metalloproteinase 9 in bone metastatic cancer cells and controls cell invasion. Mol Cell Biol 25: 8581–8591. 16166639 10.1128/MCB.25.19.8581-8591.2005 PMC1265732

[29] Akech J, Wixted JJ, Bedard K, van der Deen M, Hussain S, et al (2010) Runx2 association with progression of prostate cancer in patients: mechanisms mediating bone osteolysis and osteoblastic metastatic lesions. Oncogene 29: 811–821. 19915614 10.1038/onc.2009.389 PMC2820596

[30] Niu DF, Kondo T, Nakazawa T, Oishi N, Kawasaki T, et al (2012) Transcription factor Runx2 is a regulator of epithelial-mesenchymal transition and invasion in thyroid carcinomas. Lab Invest 92: 1181–1190. 22641097 10.1038/labinvest.2012.84

[31] Kayed H, Jiang X, Keleg S, Jesnowski R, Giese T, et al (2007) Regulation and functional role of the Runt-related transcription factor-2 in pancreatic cancer. Br J Cancer 97: 1106–1115. 17876328 10.1038/sj.bjc.6603984 PMC2360444

[32] Li W, Xu S, Lin S, Zhao W (2012) Overexpression of runt-related transcription factor-2 is associated with advanced tumor progression and poor prognosis in epithelial ovarian cancer. J Biomed Biotechnol 2012: 456534. 23093845 10.1155/2012/456534 PMC3475129

[33] Vergote I, Rustin GJ, Eisenhauer EA, Kristensen GB, Pujade-Lauraine E, et al (2000) Re: new guidelines to evaluate the response to treatment in solid tumors [ovarian cancer]. Gynecologic Cancer Intergroup. J Natl Cancer Inst 92: 1534–1535. 10995813 10.1093/jnci/92.18.1534

[34] Provencher DM, Lounis H, Champoux L, Tetrault M, Manderson EN, et al (2000) Characterization of four novel epithelial ovarian cancer cell lines. In Vitro Cell Dev Biol Anim 36: 357–361. 10949993 10.1290/1071-2690(2000)036<0357:COFNEO>2.0.CO;2

[35] Sasaki H, Sheng Y, Kotsuji F, Tsang BK (2000) Down-regulation of X-linked inhibitor of apoptosis protein induces apoptosis in chemoresistant human ovarian cancer cells. Cancer Res 60: 5659–5666. 11059757

[36] L'Esperance S, Bachvarova M, Tetu B, Mes-Masson AM, Bachvarov D (2008) Global gene expression analysis of early response to chemotherapy treatment in ovarian cancer spheroids. BMC Genomics 9: 99. 18302766 10.1186/1471-2164-9-99 PMC2279123

[37] Mercier PL, Bachvarova M, Plante M, Gregoire J, Renaud MC, et al (2011) Characterization of DOK1, a candidate tumor suppressor gene, in epithelial ovarian cancer. Mol Oncol 5: 438–453. 21856257 10.1016/j.molonc.2011.07.003 PMC5528302

[38] Keita M, Bachvarova M, Morin C, Plante M, Gregoire J, et al (2013) The RUNX1 transcription factor is expressed in serous epithelial ovarian carcinoma and contributes to cell proliferation, migration and invasion. Cell Cycle 12. 10.4161/cc.23963 PMC3637356 23442798

[39] Tetu B, Popa I, Bairati I, L'Esperance S, Bachvarova M, et al (2008) Immunohistochemical analysis of possible chemoresistance markers identified by micro-arrays on serous ovarian carcinomas. Mod Pathol 21: 1002–1010. 18500265 10.1038/modpathol.2008.80

[40] Sandi MJ, Hamidi T, Malicet C, Cano C, Loncle C, et al (2011) p8 expression controls pancreatic cancer cell migration, invasion, adhesion, and tumorigenesis. J Cell Physiol 226: 3442–3451. 21344397 10.1002/jcp.22702

[41] Cano CE, Hamidi T, Sandi MJ, Iovanna JL (2011) Nupr1: the Swiss-knife of cancer. J Cell Physiol 226: 1439–1443. 20658514 10.1002/jcp.22324

[42] Kanamori Y, Matsushima M, Minaguchi T, Kobayashi K, Sagae S, et al (1999) Correlation between expression of the matrix metalloproteinase-1 gene in ovarian cancers and an insertion/deletion polymorphism in its promoter region. Cancer Res 59: 4225–4227. 10485461

[43] Zhao H, Yang Z, Wang X, Zhang X, Wang M, et al (2012) Triptolide inhibits ovarian cancer cell invasion by repression of matrix metalloproteinase 7 and 19 and upregulation of E-cadherin. Exp Mol Med 44: 633–641. 22902510 10.3858/emm.2012.44.11.072 PMC3509180

[44] Daikoku T, Wang D, Tranguch S, Morrow JD, Orsulic S, et al (2005) Cyclooxygenase-1 is a potential target for prevention and treatment of ovarian epithelial cancer. Cancer Res 65: 3735–3744. 15867369 10.1158/0008-5472.CAN-04-3814 PMC2584020

[45] Purcell DJ, Khalid O, Ou CY, Little GH, Frenkel B, et al (2012) Recruitment of coregulator G9a by Runx2 for selective enhancement or suppression of transcription. J Cell Biochem 113: 2406–2414. 22389001 10.1002/jcb.24114 PMC3350606

[46] van der Deen M, Akech J, Lapointe D, Gupta S, Young DW, et al (2012) Genomic promoter occupancy of runt-related transcription factor RUNX2 in Osteosarcoma cells identifies genes involved in cell adhesion and motility. J Biol Chem 287: 4503–4517. 22158627 10.1074/jbc.M111.287771 PMC3281617

[47] Li H, Zhou RJ, Zhang GQ, Xu JP (2013) Clinical significance of RUNX2 expression in patients with nonsmall cell lung cancer: a 5-year follow-up study. Tumour Biol 10.1007/s13277-013-0720-4 23471668

[48] Kim DJ, Akiyama TE, Harman FS, Burns AM, Shan W, et al (2004) Peroxisome proliferator-activated receptor beta (delta)-dependent regulation of ubiquitin C expression contributes to attenuation of skin carcinogenesis. J Biol Chem 279: 23719–23727. 15033975 10.1074/jbc.M312063200

[49] Huang Z, Cheng Y, Chiu PM, Cheung FM, Nicholls JM, et al (2012) Tumor suppressor Alpha B-crystallin (CRYAB) associates with the cadherin/catenin adherens junction and impairs NPC progression-associated properties. Oncogene 31: 3709–3720. 22158051 10.1038/onc.2011.529

[50] Fridman JS, Rehemtulla A, Hofmann A, Blau HM, Maybaum J (1998) Expression of Bcl-XS alters cytokinetics and decreases clonogenic survival in K12 rat colon carcinoma cells. Oncogene 17: 2981–2991. 9881700 10.1038/sj.onc.1202224

[51] Mansour M, Palese P, Zamarin D (2011) Oncolytic specificity of Newcastle disease virus is mediated by selectivity for apoptosis-resistant cells. J Virol 85: 6015–6023. 21471241 10.1128/JVI.01537-10 PMC3126310

[52] Hishikawa K, Oemar BS, Tanner FC, Nakaki T, Luscher TF, et al (1999) Connective tissue growth factor induces apoptosis in human breast cancer cell line MCF-7. J Biol Chem 274: 37461–37466. 10601320 10.1074/jbc.274.52.37461

[53] Capparelli C, Whitaker-Menezes D, Guido C, Balliet R, Pestell TG, et al (2012) CTGF drives autophagy, glycolysis and senescence in cancer-associated fibroblasts via HIF1 activation, metabolically promoting tumor growth. Cell Cycle 11: 2272–2284. 22684333 10.4161/cc.20717 PMC3383589

[54] Yang MH, Lin BR, Chang CH, Chen ST, Lin SK, et al (2012) Connective tissue growth factor modulates oral squamous cell carcinoma invasion by activating a miR-504/FOXP1 signalling. Oncogene 31: 2401–2411. 21927029 10.1038/onc.2011.423

[55] Ofek P, Ben-Meir D, Kariv-Inbal Z, Oren M, Lavi S (2003) Cell cycle regulation and p53 activation by protein phosphatase 2C alpha. J Biol Chem 278: 14299–14305. 12514180 10.1074/jbc.M211699200

[56] Morrow KA, Das S, Metge BJ, Ye K, Mulekar MS, et al (2011) Loss of tumor suppressor Merlin in advanced breast cancer is due to post-translational regulation. J Biol Chem 286: 40376–40385. 21965655 10.1074/jbc.M111.250035 PMC3220570

[57] Li W, Giancotti FG (2010) Merlin's tumor suppression linked to inhibition of the E3 ubiquitin ligase CRL4 (DCAF1). Cell Cycle 9: 4433–4436. 21084862 10.4161/cc.9.22.13838 PMC3048042

[58] Lecomte C, Andujar P, Renier A, Kheuang L, Abramowski V, et al (2005) Similar tumor suppressor gene alteration profiles in asbestos-induced murine and human mesothelioma. Cell Cycle 4: 1862–1869. 16319530 10.4161/cc.4.12.2300

[59] Daikoku T, Tranguch S, Chakrabarty A, Wang D, Khabele D, et al (2007) Extracellular signal-regulated kinase is a target of cyclooxygenase-1-peroxisome proliferator-activated receptor-delta signaling in epithelial ovarian cancer. Cancer Res 67: 5285–5292. 17545608 10.1158/0008-5472.CAN-07-0828

[60] Madsen CV, Steffensen KD, Olsen DA, Waldstrom M, Sogaard CH, et al (2012) Serum platelet-derived growth factor and fibroblast growth factor in patients with benign and malignant ovarian tumors. Anticancer Res 32: 3817–3825. 22993324

[61] Papacleovoulou G, Critchley HO, Hillier SG, Mason JI (2011) IL1alpha and IL4 signalling in human ovarian surface epithelial cells. J Endocrinol 211: 273–283. 21903865 10.1530/JOE-11-0081

[62] Brard L, Lange TS, Robison K, Kim KK, Ara T, et al (2011) Evaluation of the first Ergocalciferol-derived, non hypercalcemic anti-cancer agent MT19c in ovarian cancer SKOV-3 cell lines. Gynecol Oncol 123: 370–378. 21803404 10.1016/j.ygyno.2011.07.002

[63] Sundfeldt K, Ivarsson K, Carlsson M, Enerback S, Janson PO, et al (1999) The expression of CCAAT/enhancer binding protein (C/EBP) in the human ovary in vivo: specific increase in C/EBPbeta during epithelial tumour progression. Br J Cancer 79: 1240–1248. 10098766 10.1038/sj.bjc.6690199 PMC2362217

[64] Mann AP, Somasunderam A, Nieves-Alicea R, Li X, Hu A, et al (2010) Identification of thioaptamer ligand against E-selectin: potential application for inflamed vasculature targeting. PLoS One 5. 10.1371/journal.pone.0013050 PMC2948018 20927342

[65] Stone B, Schummer M, Paley PJ, Thompson L, Stewart J, et al (2003) Serologic analysis of ovarian tumor antigens reveals a bias toward antigens encoded on 17q. Int J Cancer 104: 73–84. 12532422 10.1002/ijc.10900

[66] Lu KH, Patterson AP, Wang L, Marquez RT, Atkinson EN, et al (2004) Selection of potential markers for epithelial ovarian cancer with gene expression arrays and recursive descent partition analysis. Clin Cancer Res 10: 3291–3300. 15161682 10.1158/1078-0432.CCR-03-0409

[67] Li H, Bitler BG, Vathipadiekal V, Maradeo ME, Slifker M, et al (2012) ALDH1A1 is a novel EZH2 target gene in epithelial ovarian cancer identified by genome-wide approaches. Cancer Prev Res (Phila) 5: 484–491. 22144423 10.1158/1940-6207.CAPR-11-0414 PMC3294119

[68] Bock AJ, Stavnes HT, Kempf T, Trope CG, Berner A, et al (2010) Expression and clinical role of growth differentiation factor-15 in ovarian carcinoma effusions. Int J Gynecol Cancer 20: 1448–1455. 21336029 10.1111/IGC.0b013e3181f7d6be

[69] Agarwal A, Tressel SL, Kaimal R, Balla M, Lam FH, et al (2010) Identification of a metalloprotease-chemokine signaling system in the ovarian cancer microenvironment: implications for antiangiogenic therapy. Cancer Res 70: 5880–5890. 20570895 10.1158/0008-5472.CAN-09-4341 PMC2917243

[70] Hantke B, Harbeck N, Schmalfeldt B, Claes I, Hiller O, et al (2003) Clinical relevance of matrix metalloproteinase-13 determined with a new highly specific and sensitive ELISA in ascitic fluid of advanced ovarian carcinoma patients. Biol Chem 384: 1247–1251. 12974393 10.1515/BC.2003.137

[71] Alper O, Bergmann-Leitner ES, Abrams S, Cho-Chung YS (2001) Apoptosis, growth arrest and suppression of invasiveness by CRE-decoy oligonucleotide in ovarian cancer cells: protein kinase A downregulation and cytoplasmic export of CRE-binding proteins. Mol Cell Biochem 218: 55–63. 11330838 10.1023/a:1007205205131

[72] Casaburi I, Avena P, Lanzino M, Sisci D, Giordano F, et al (2012) Chenodeoxycholic acid through a TGR5-dependent CREB signaling activation enhances cyclin D1 expression and promotes human endometrial cancer cell proliferation. Cell Cycle 11: 2699–2710. 22751440 10.4161/cc.21029

[73] Kinjo K, Sandoval S, Sakamoto KM, Shankar DB (2005) The role of CREB as a proto-oncogene in hematopoiesis. Cell Cycle 4: 1134–1135. 16096372 10.4161/cc.4.9.1991

[74] Duprez E (2004) A new role for C/EBPbeta in acute promyelocytic leukemia. Cell Cycle 3: 389–390. 14976428 10.4161/cc.3.4.771

[75] Tamura K, Hua B, Adachi S, Guney I, Kawauchi J, et al (2005) Stress response gene ATF3 is a target of c-myc in serum-induced cell proliferation. EMBO J 24: 2590–2601. 15990869 10.1038/sj.emboj.7600742 PMC1176468

[76] Lehtinen L, Ketola K, Makela R, Mpindi JP, Viitala M, et al (2013) High-throughput RNAi screening for novel modulators of vimentin expression identifies MTHFD2 as a regulator of breast cancer cell migration and invasion. Oncotarget 4: 48–63. 23295955 10.18632/oncotarget.756 PMC3702207

[77] Taraboletti G, Rusnati M, Ragona L, Colombo G (2010) Targeting tumor angiogenesis with TSP-1-based compounds: rational design of antiangiogenic mimetics of endogenous inhibitors. Oncotarget 1: 662–673. 21317461 10.18632/oncotarget.200 PMC3248139

[78] Karnezis T, Shayan R, Fox S, Achen MG, Stacker SA (2012) The connection between lymphangiogenic signalling and prostaglandin biology: a missing link in the metastatic pathway. Oncotarget 3: 893–906. 23097685 10.18632/oncotarget.593 PMC3478465

[79] Yamaoka T, Fujimoto M, Ogawa F, Yoshizaki A, Bae SJ, et al (2011) The roles of P- and E-selectins and P-selectin glycoprotein ligand-1 in primary and metastatic mouse melanomas. J Dermatol Sci 64: 99–107. 21889879 10.1016/j.jdermsci.2011.07.005

[80] Okada-Ban M, Moens G, Thiery JP, Jouanneau J (1999) Nuclear 24 kD fibroblast growth factor (FGF)-2 confers metastatic properties on rat bladder carcinoma cells. Oncogene 18: 6719–6724. 10597279 10.1038/sj.onc.1203092

[81] Nakanishi K, Yoshimoto T, Tsutsui H, Okamura H (2001) Interleukin-18 is a unique cytokine that stimulates both Th1 and Th2 responses depending on its cytokine milieu. Cytokine Growth Factor Rev 12: 53–72. 11312119 10.1016/s1359-6101(00)00015-0

[82] Yan C, Boyd DD (2006) ATF3 regulates the stability of p53: a link to cancer. Cell Cycle 5: 926–929. 16628010 10.4161/cc.5.9.2714

[83] Sternlicht MD, Werb Z (2001) How matrix metalloproteinases regulate cell behavior. Annu Rev Cell Dev Biol 17: 463–516. 11687497 10.1146/annurev.cellbio.17.1.463 PMC2792593

