# Supplementary material for: Inhibition of RUNX2 Transcriptional Activity Blocks the Proliferation, Migration and Invasion of Epithelial Ovarian Carcinoma Cells
Source: PLoS One. 2013 Oct 4;8(10):e74384. doi: 10.1371/journal.pone.0074384 (PMC3790792; doi:10.1371/journal.pone.0074384)
Supplement: Figure S1 — Kaplan-Meier curve for progression free survival according to the level of RUNX2 IHC intensity in tumor samples of 52 serous EOC patients. (PPT) [file pone.0074384.s001.ppt]

## Slide 1
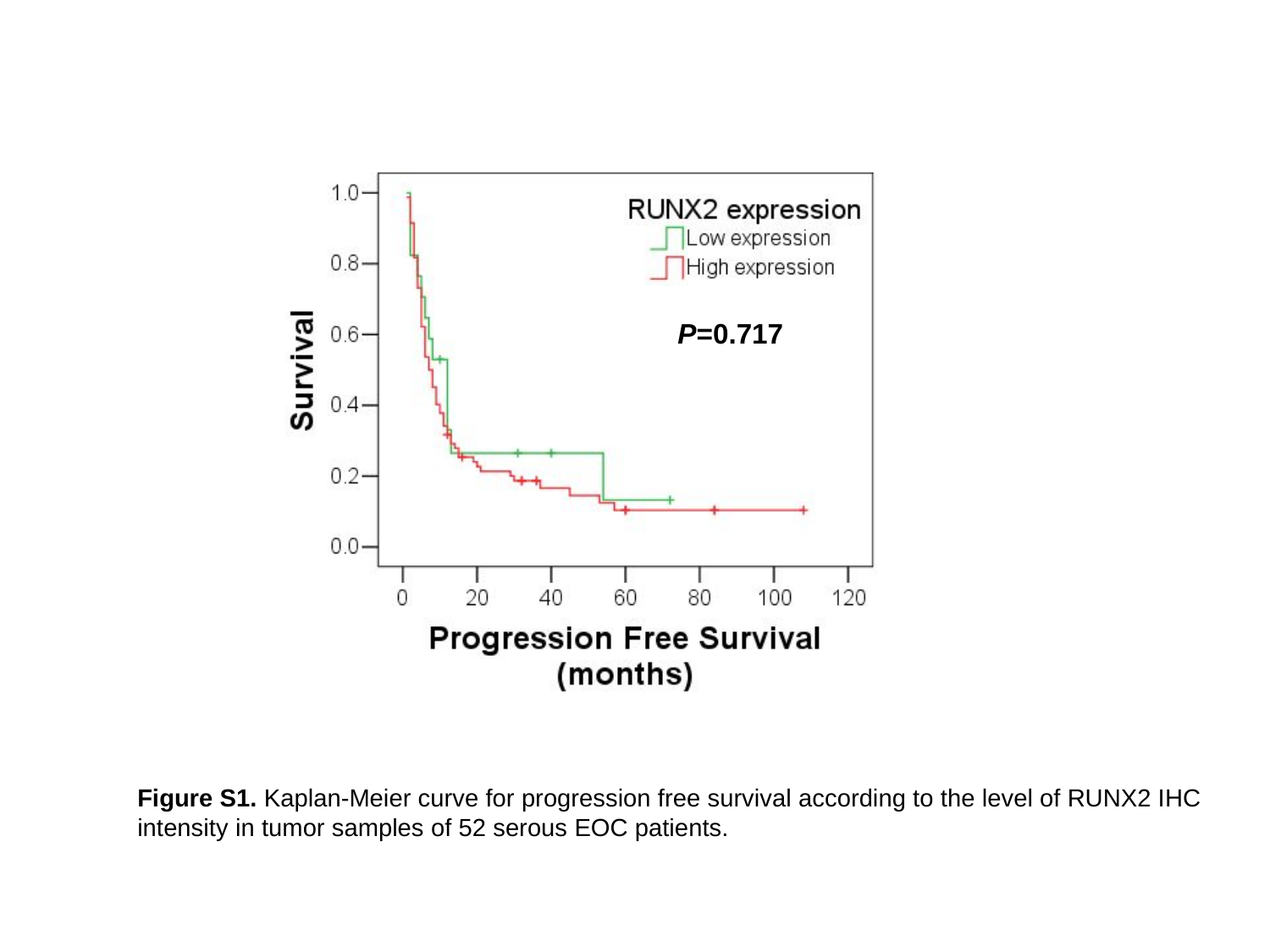

P=0.717
Figure S1. Kaplan-Meier curve for progression free survival according to the level of RUNX2 IHC intensity in tumor samples of 52 serous EOC patients.
