# Supplementary material for: Inhibition of RUNX2 Transcriptional Activity Blocks the Proliferation, Migration and Invasion of Epithelial Ovarian Carcinoma Cells
Source: PLoS One. 2013 Oct 4;8(10):e74384. doi: 10.1371/journal.pone.0074384 (PMC3790792; doi:10.1371/journal.pone.0074384)
Supplement: Figure S2 — Genomic structure of the RUNX2 gene, isoforms a, b and c. The CpG island (CpG 60) containing the analyzed 12 putative CpG methylation targets is indicated with arrow. (PPT) [file pone.0074384.s002.ppt]

## Slide 1
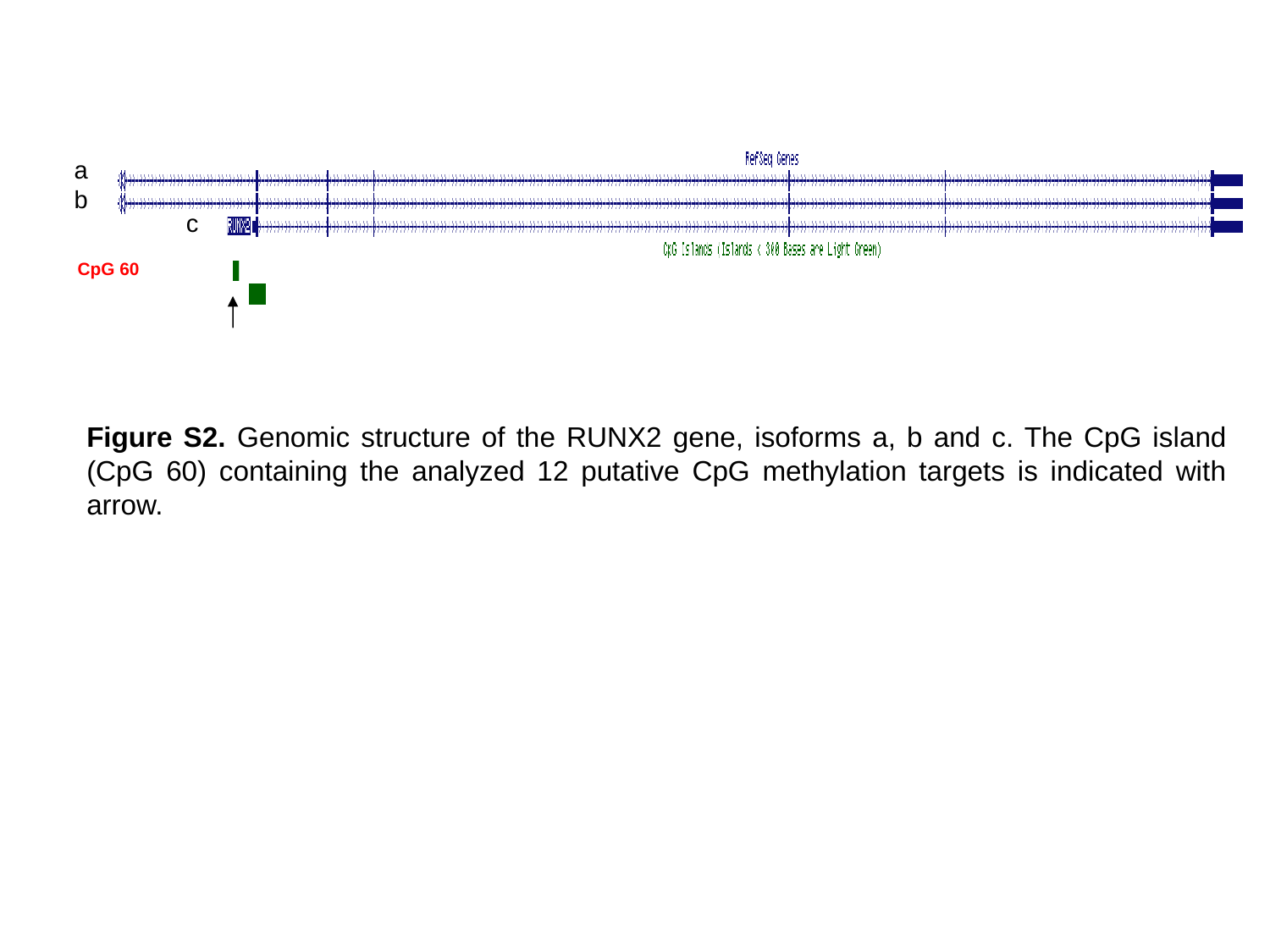

a
b
c
CpG 60
Figure S2. Genomic structure of the RUNX2 gene, isoforms a, b and c. The CpG island (CpG 60) containing the analyzed 12 putative CpG methylation targets is indicated with arrow.
