# Supplementary material for: Inhibition of RUNX2 Transcriptional Activity Blocks the Proliferation, Migration and Invasion of Epithelial Ovarian Carcinoma Cells
Source: PLoS One. 2013 Oct 4;8(10):e74384. doi: 10.1371/journal.pone.0074384 (PMC3790792; doi:10.1371/journal.pone.0074384)
Supplement: Figure S3 — BSP analysis of the methylation status of RUNX2 in grade 3 primary serous EOC tumors compared to omental metastases. (PPT) [file pone.0074384.s003.ppt]

## Slide 1
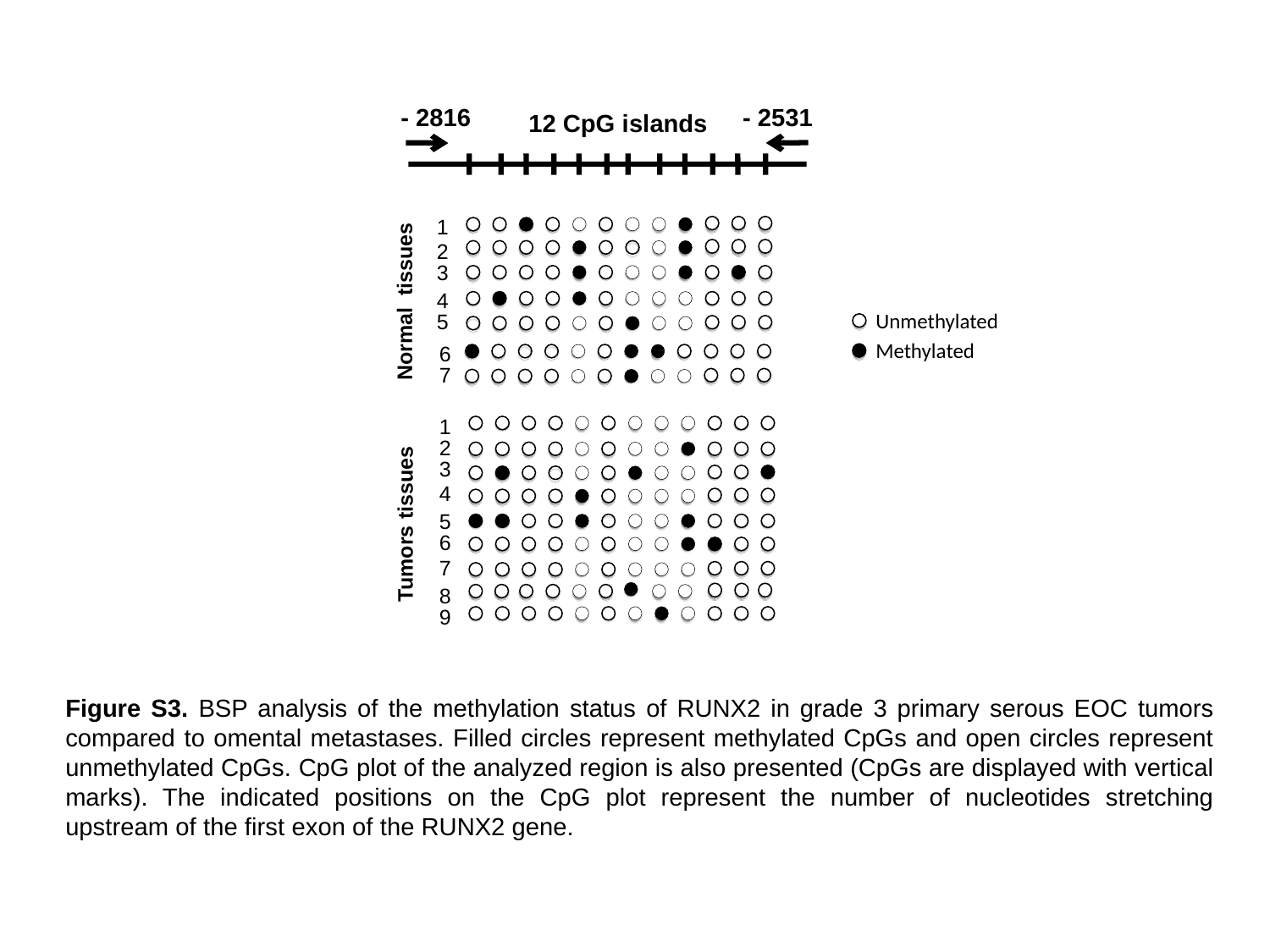

- 2816
- 2531
12 CpG islands
1
2
3
Normal tissues
4
Unmethylated
5
Methylated
6
7
1
2
3
4
Tumors tissues
5
6
7
8
9
Figure S3. BSP analysis of the methylation status of RUNX2 in grade 3 primary serous EOC tumors compared to omental metastases. Filled circles represent methylated CpGs and open circles represent unmethylated CpGs. CpG plot of the analyzed region is also presented (CpGs are displayed with vertical marks). The indicated positions on the CpG plot represent the number of nucleotides stretching upstream of the first exon of the RUNX2 gene.
