# Supplementary material for: Inhibition of RUNX2 Transcriptional Activity Blocks the Proliferation, Migration and Invasion of Epithelial Ovarian Carcinoma Cells
Source: PLoS One. 2013 Oct 4;8(10):e74384. doi: 10.1371/journal.pone.0074384 (PMC3790792; doi:10.1371/journal.pone.0074384)
Supplement: Figure S4 — Western blot analysis of RUNX2 protein expression in different EOC cell lines. (PPT) [file pone.0074384.s004.ppt]

## Slide 1
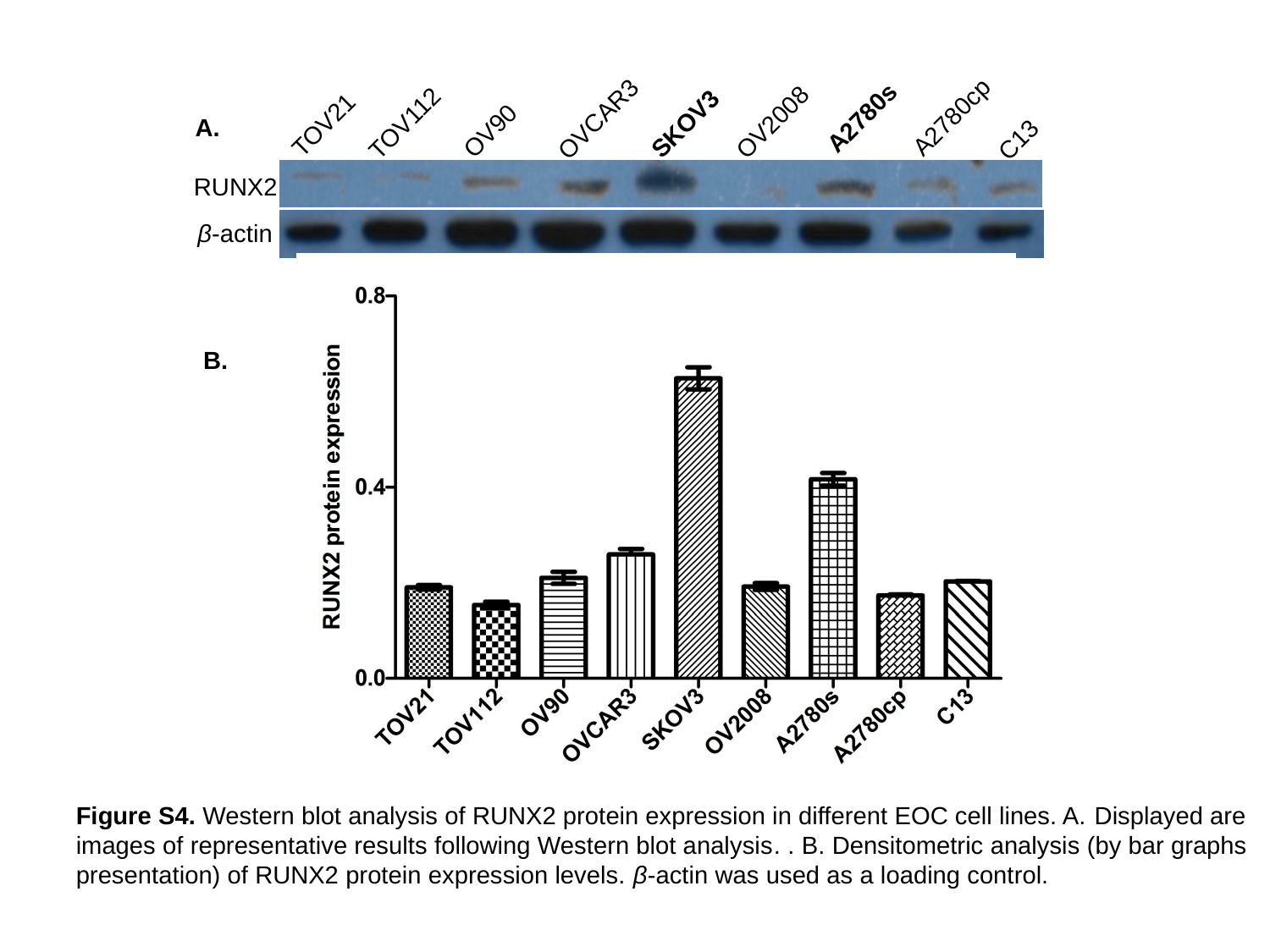

A2780cp
A2780s
OVCAR3
SKOV3
OV2008
TOV112
TOV21
OV90
C13
RUNX2
β-actin
A.
B.
Figure S4. Western blot analysis of RUNX2 protein expression in different EOC cell lines. A. Displayed are images of representative results following Western blot analysis. . B. Densitometric analysis (by bar graphs presentation) of RUNX2 protein expression levels. β-actin was used as a loading control.
