# Supplementary material for: Inhibition of RUNX2 Transcriptional Activity Blocks the Proliferation, Migration and Invasion of Epithelial Ovarian Carcinoma Cells
Source: PLoS One. 2013 Oct 4;8(10):e74384. doi: 10.1371/journal.pone.0074384 (PMC3790792; doi:10.1371/journal.pone.0074384)
Supplement: Figure S5 — ShRNA-mediated knockdown of the RUNX2 expression in A2780s cells and consecutive analyses of functional phenotypes. (PPT) [file pone.0074384.s005.ppt]

## Slide 1
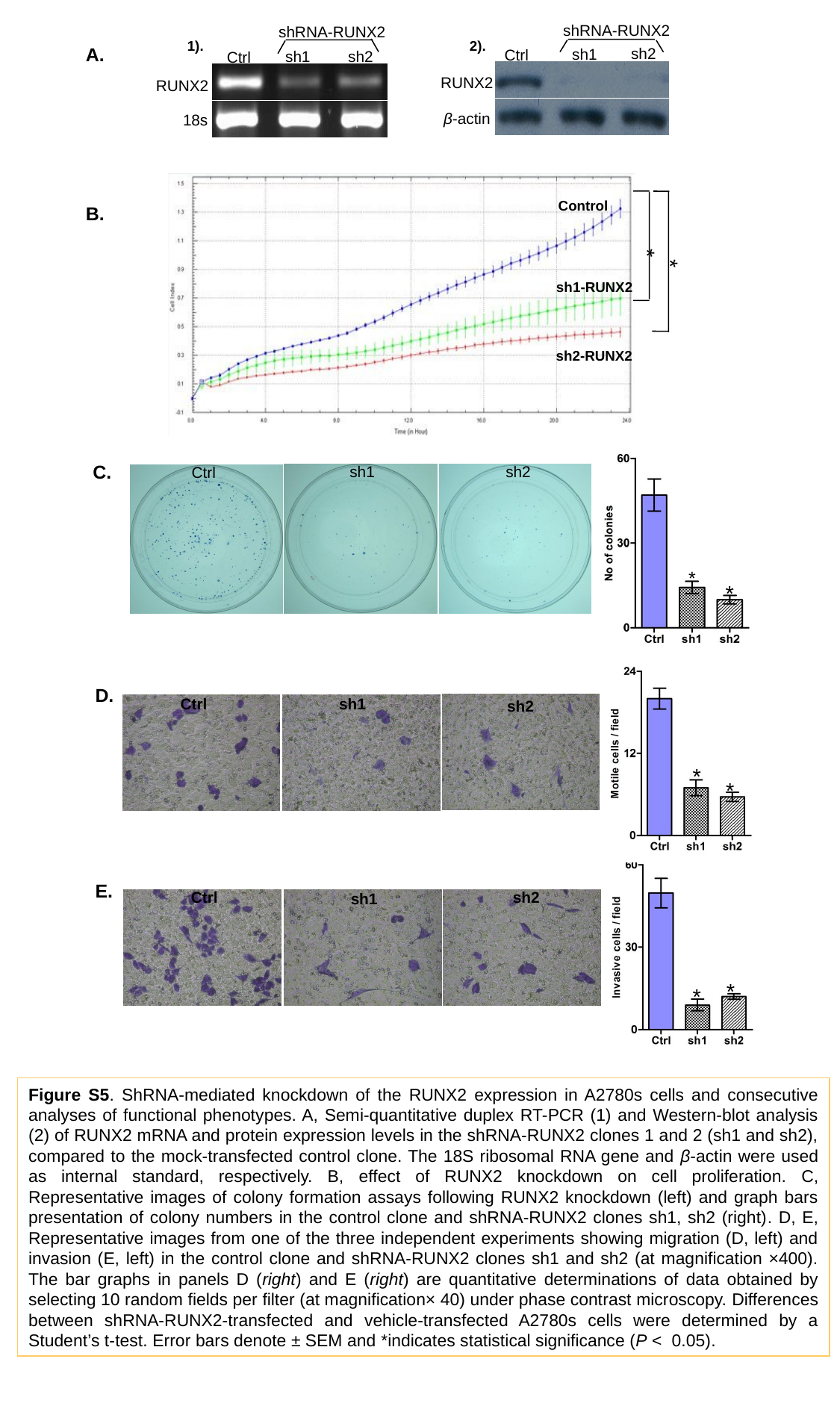

shRNA-RUNX2
sh2
sh1
Ctrl
RUNX2
 β-actin
shRNA-RUNX2
sh1
sh2
Ctrl
RUNX2
 18s
1).
2).
A.
Control
*
sh1-RUNX2
*
sh2-RUNX2
B.
*
*
**
**
*
*
 *
**
sh1
sh2
Ctrl
*
*
 *
**
C.
*
*
Ctrl
sh1
sh2
D.
*
*
Ctrl
sh2
sh1
E.
Figure S5. ShRNA-mediated knockdown of the RUNX2 expression in A2780s cells and consecutive analyses of functional phenotypes. A, Semi-quantitative duplex RT-PCR (1) and Western-blot analysis (2) of RUNX2 mRNA and protein expression levels in the shRNA-RUNX2 clones 1 and 2 (sh1 and sh2), compared to the mock-transfected control clone. The 18S ribosomal RNA gene and β-actin were used as internal standard, respectively. B, effect of RUNX2 knockdown on cell proliferation. C, Representative images of colony formation assays following RUNX2 knockdown (left) and graph bars presentation of colony numbers in the control clone and shRNA-RUNX2 clones sh1, sh2 (right). D, E, Representative images from one of the three independent experiments showing migration (D, left) and invasion (E, left) in the control clone and shRNA-RUNX2 clones sh1 and sh2 (at magnification ×400). The bar graphs in panels D (right) and E (right) are quantitative determinations of data obtained by selecting 10 random fields per filter (at magnification× 40) under phase contrast microscopy. Differences between shRNA-RUNX2-transfected and vehicle-transfected A2780s cells were determined by a Student’s t-test. Error bars denote ± SEM and *indicates statistical significance (P < 0.05).
