# Supplementary material for: Inhibition of RUNX2 Transcriptional Activity Blocks the Proliferation, Migration and Invasion of Epithelial Ovarian Carcinoma Cells
Source: PLoS One. 2013 Oct 4;8(10):e74384. doi: 10.1371/journal.pone.0074384 (PMC3790792; doi:10.1371/journal.pone.0074384)
Supplement: Figure S6 — ShRNA-mediated knockdown of the RUNX2 expression in SKOV3 cells: effect on cell cycle control. (PPT) [file pone.0074384.s006.ppt]

## Slide 1
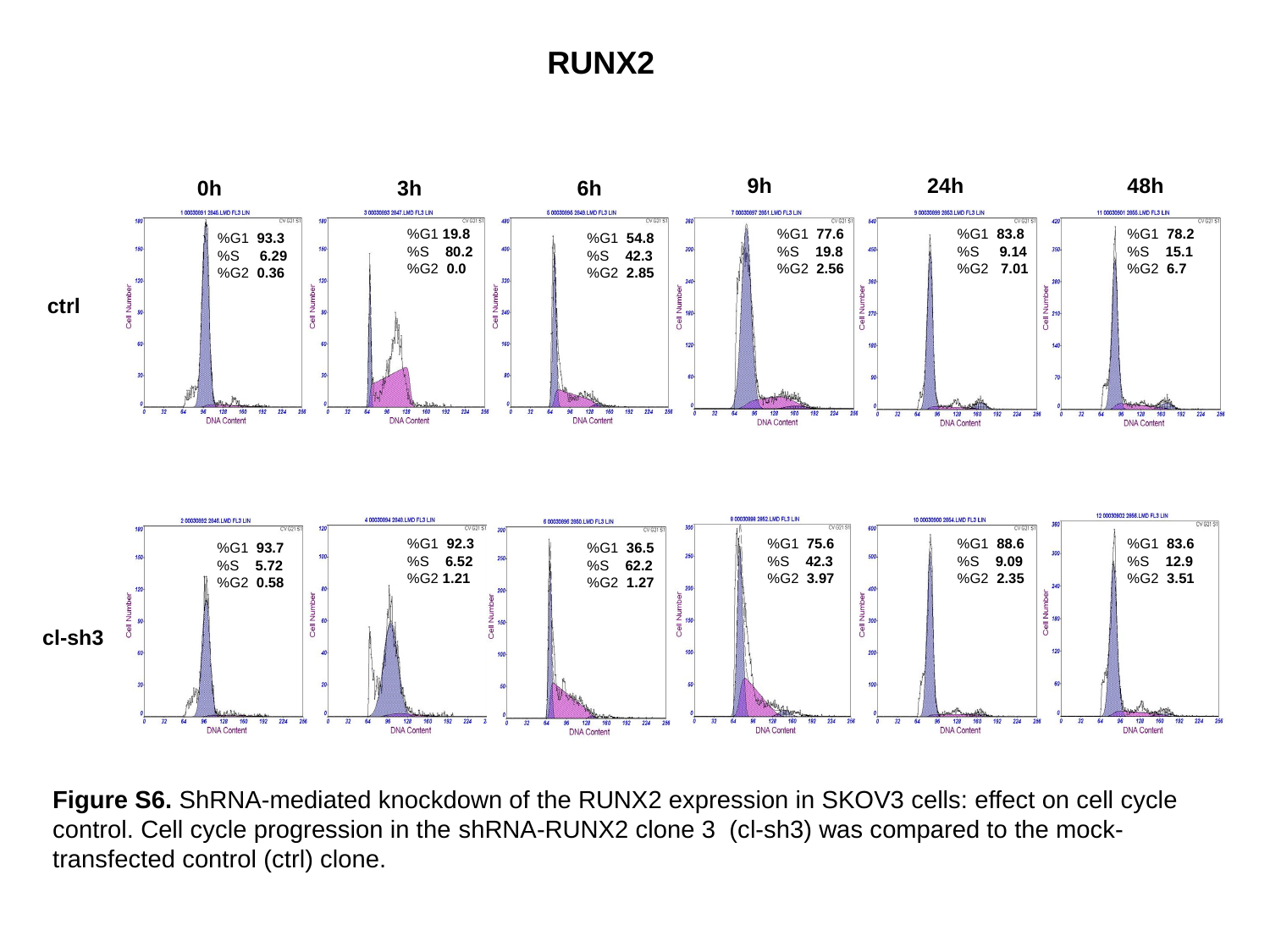

RUNX2
9h
24h
48h
0h
3h
6h
%G1 19.8
%S 80.2
%G2 0.0
%G1 77.6
%S 19.8
%G2 2.56
%G1 83.8
%S 9.14
%G2 7.01
%G1 78.2
%S 15.1
%G2 6.7
%G1 93.3
%S 6.29
%G2 0.36
%G1 54.8
%S 42.3
%G2 2.85
ctrl
%G1 92.3
%S 6.52
%G2 1.21
%G1 75.6
%S 42.3
%G2 3.97
%G1 88.6
%S 9.09
%G2 2.35
%G1 83.6
%S 12.9
%G2 3.51
%G1 93.7
%S 5.72
%G2 0.58
%G1 36.5
%S 62.2
%G2 1.27
cl-sh3
Figure S6. ShRNA-mediated knockdown of the RUNX2 expression in SKOV3 cells: effect on cell cycle control. Cell cycle progression in the shRNA-RUNX2 clone 3 (cl-sh3) was compared to the mock-transfected control (ctrl) clone.
