# Supplementary material for: Inhibition of RUNX2 Transcriptional Activity Blocks the Proliferation, Migration and Invasion of Epithelial Ovarian Carcinoma Cells
Source: PLoS One. 2013 Oct 4;8(10):e74384. doi: 10.1371/journal.pone.0074384 (PMC3790792; doi:10.1371/journal.pone.0074384)
Supplement: Figure S7 — Dose-response cytotoxicity curves upon cisplatin (A) and paclitaxel (B) treatment of SKOV3 cells following shRNA-mediated RUNX2 knockdown. (PPT) [file pone.0074384.s007.ppt]

## Slide 1
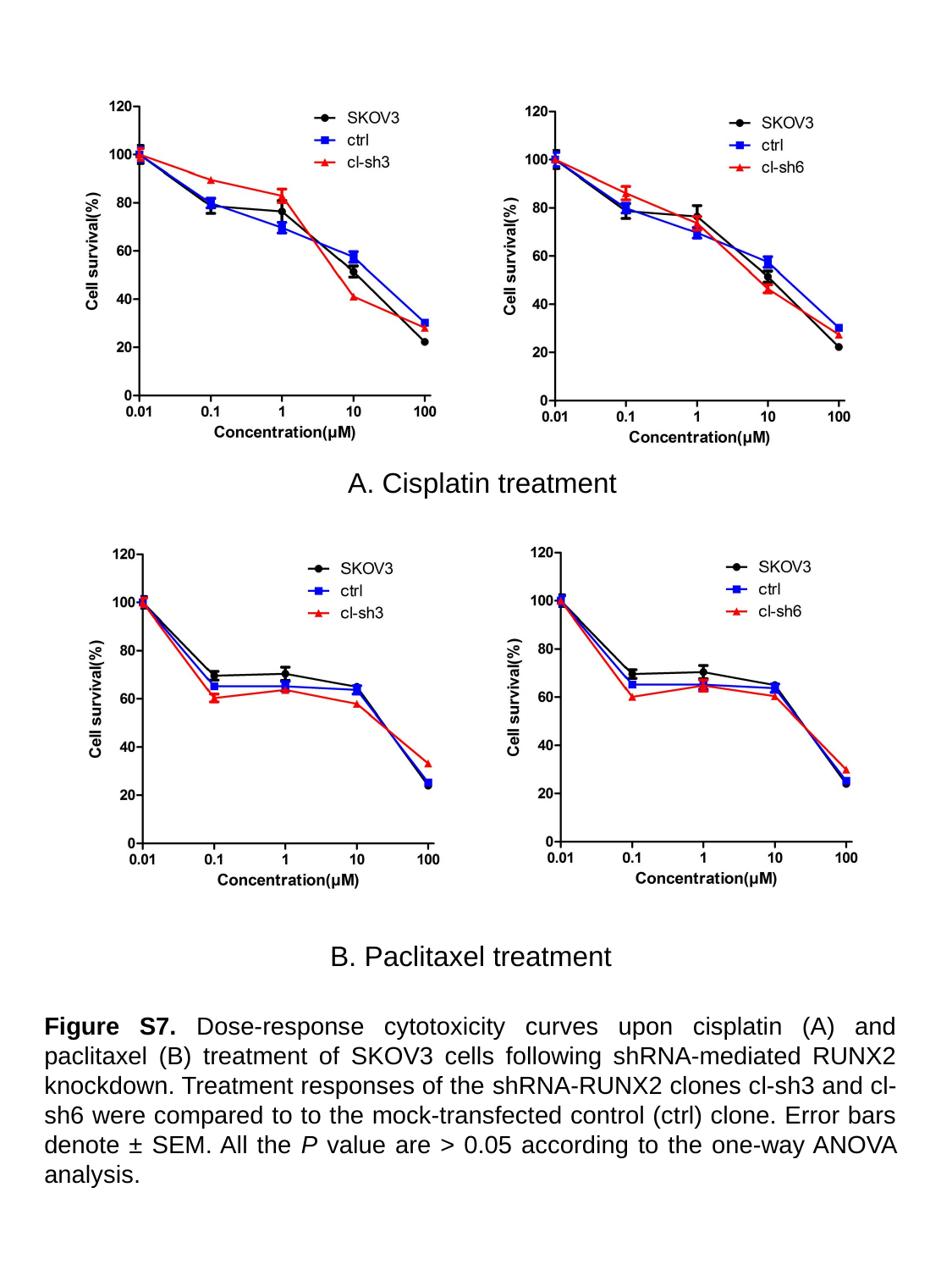

A. Cisplatin treatment
B. Paclitaxel treatment
Figure S7. Dose-response cytotoxicity curves upon cisplatin (A) and paclitaxel (B) treatment of SKOV3 cells following shRNA-mediated RUNX2 knockdown. Treatment responses of the shRNA-RUNX2 clones cl-sh3 and cl-sh6 were compared to to the mock-transfected control (ctrl) clone. Error bars denote ± SEM. All the P value are > 0.05 according to the one-way ANOVA analysis.
