# Supplementary material for: Inhibition of RUNX2 Transcriptional Activity Blocks the Proliferation, Migration and Invasion of Epithelial Ovarian Carcinoma Cells
Source: PLoS One. 2013 Oct 4;8(10):e74384. doi: 10.1371/journal.pone.0074384 (PMC3790792; doi:10.1371/journal.pone.0074384)
Supplement: Table S4 — Median comparison analysis (Mann-Whitney) of the RUNX2 expression values in different EOC and control tissues. (DOC) [file pone.0074384.s011.doc]

**Table S4.** Median comparison analysis (Mann-Whitney) of the RUNX2 expression values in different EOC and control tissues, including 52 primary high-grade tumors (HG), 52 omental metastases (OM), 13 low-malignant potential tumors (LMP), 13 ovarian normal tissues (ONT) and 13 uterine smooth muscle (SMT) tissues (as presented in Fig. 1).

| Tissue | Mean + SD | Median | Lower 95% CI | Upper 95% CI |
| --- | --- | --- | --- | --- |
| **SMT** | 0,54±0,37 | 0,50 | 0,33 | 0,75 |
| **ONT** | 1,11±0,60 | 1,00 | 0,65 | 1,57 |
| **LMP** | 2,48±0,44 | 2,50 | 2,21 | 2,74 |
| **HG** | 2,29±0,77 | 2,50 | 2,01 | 2,51 |
| **OM** | 2,07±0,62 | 2,00 | 1,90 | 2,24 |
